# Gender-associated cardiometabolic risk profiles and health behaviors in patients with type 2 diabetes: a cross-sectional analysis of the Joint Asia Diabetes Evaluation (JADE) program

**DOI:** 10.1016/j.lanwpc.2022.100663

**Published:** 2022-12-19

**Authors:** Lee-Ling Lim, Eric S.H. Lau, Alice P.S. Kong, Amy W.C. Fu, Vanessa Lau, Weiping Jia, Wayne H.H. Sheu, Leorino Sobrepena, K.H. Yoon, Alexander T.B. Tan, Yook-Chin Chia, Aravind Sosale, Banshi D. Saboo, Jothydev Kesavadev, Su-Yen Goh, Thy Khue Nguyen, Yotsapon Thewjitcharoen, Raymond Suwita, Ronald C.W. Ma, Elaine Y.K. Chow, Andrea O.Y. Luk, Juliana C.N. Chan

**Affiliations:** a Department of Medicine, Faculty of Medicine, University of Malaya, Kuala Lumpur, Malaysia; b Department of Medicine and Therapeutics, The Chinese University of Hong Kong, Prince of Wales Hospital, Hong Kong Special Administrative Region, China; c Asia Diabetes Foundation, Shatin, Hong Kong Special Administrative Region, China; d Hong Kong Institute of Diabetes and Obesity, The Chinese University of Hong Kong, Prince of Wales Hospital, Hong Kong Special Administrative Region, China; e Li Ka Shing Institute of Health Sciences, The Chinese University of Hong Kong, Prince of Wales Hospital, Hong Kong Special Administrative Region, China; f Department of Endocrinology and Metabolism, Shanghai Clinical Centre for Diabetes, Shanghai Diabetes Institute, Shanghai Key Laboratory of Diabetes Mellitus, Shanghai Jiao Tong University Affiliated Sixth People's Hospital, Shanghai, China; g Department of Medicine, Taipei Veterans General Hospital, Taipei, Taiwan; h Heart of Jesus Hospital, San Jose City, Philippines; i Department of Endocrinology and Metabolism, Seoul St. Mary's Hospital, The Catholic University of Korea, Seoul, Korea; j Sunway Medical Centre, Selangor, Malaysia; k Department of Medical Sciences, School of Medical and Life Sciences, Sunway University, Selangor, Malaysia; l Department of Primary Care Medicine, Faculty of Medicine, University of Malaya, Kuala Lumpur, Malaysia; m Diacon Hospital, Bangalore, India; n Dia Care - Diabetes Care & Hormone Clinic, Gujarat, India; o Jothydev's Diabetes and Research Center, Kerala, India; p Department of Endocrinology, Singapore General Hospital, Singapore; q MEDIC Medical Centre, Ho Chi Minh City, Vietnam; r Diabetes and Thyroid Center, Theptarin Hospital, Bangkok, Thailand; s Cerebrocardiovascular Diabetes Group Clinic (CDG), Jakarta, Indonesia

**Keywords:** Disparity, Care gaps, Inequality, Ethnicity, Type 2 diabetes, Treatment targets, Cardiovascular risk factors, Comorbidity, Self-management

## Abstract

**Background:**

In Asia, diabetes-associated death due to cardiorenal diseases were 2–3 times higher in women than men which might be due to gender disparity in quality of care and health habits.

**Methods:**

Adults with type 2 diabetes (T2D) from 11 Asian countries/areas were assessed using the same protocol (2007–2015). We compared treatment target attainment (HbA_1c_ < 7%, blood pressure [BP] < 130/80 mmHg, risk-based LDL-cholesterol, lack of central obesity [waist circumference <90 cm in men or <80 cm in women), use of cardiorenal-protective drugs (renin-angiotensin system [RAS] inhibitors, statins), and self-reported health habits including self-monitoring blood glucose (SMBG) by gender. Analyses were stratified by countries/areas, age of natural menopause (<50 *vs.* ≥50 years), and comorbidities (atherosclerotic cardiovascular disease [ASCVD], heart failure, kidney impairment [eGFR < 60 mL/min/1.73 m^2^]).

**Findings:**

Among 106,376 patients (53.2% men; median (interquartile range) diabetes duration: 6.0 (2.0–12.0) years; mean ± SD HbA_1c_ 8.0 ± 1.9%; 27% insulin-treated), women were older and less likely to receive college education than men (28.9% *vs.* 48.8%). Women were less likely to smoke/drink alcohol and were physically less active than men. Women had lower BP (<130/80 mmHg: 29.4% *vs.* 25.7%), less general obesity (54.8% *vs.* 57.8%) but more central obesity than men (77.5% *vs.* 57.3%). Women were less likely to have ASCVD (12.8% *vs.* 17.0%) or heart failure (1.3% *vs.* 2.3%), but more likely to have kidney impairment (22.3% *vs.* 17.6%) and any-site cancer than men (2.5% *vs.* 1.6%). In most countries/areas, more men attained HbA_1c_ <7% and risk-based LDL-cholesterol level than women. After adjusting for potential confounders including countries and centres, men had 1.63 odds ratio (95% CI 1.51, 1.74) of attaining ≥3 treatment targets than women.

**Interpretation:**

Asian women with T2D had worse quality of care than men especially in middle-income countries/areas, calling for targeted implementation programs to close these care gaps.

**Sponsor:**

Asia Diabetes Foundation.

**Funding:**

Nil.

**Research in context**

**Evidence before this study**

Asia has the largest number of people with diabetes characterised by diverse genetic, lifecourse, and demographic profiles with considerable heterogeneity in educational levels, health literacy, health systems, and access to care. In Asia, women with diabetes were 2–3 times more likely to die from cardiovascular-renal disease than men with diabetes after age adjustment. We searched PubMed for articles published from inception to October 24, 2022, using the Medical Subject Headings (MeSH) search terms “gender”, “disparity”, “Asia”, and “type 2 diabetes” with no language restrictions. We identified 50 studies but none of them reported gender differences in demographics, risk factors, comorbidities, and quality of care among people with type 2 diabetes (T2D) across Asia.

**Added value of this study**

In Asia, women attending clinics in 11 countries/areas including China, Hong Kong, Taiwan, Singapore, Korea, Vietnam, Thailand, Indonesia, India, Malaysia, and Philippines exhibited disparity in demographics, health habits, and quality of care. Women were more likely to have central obesity, physical inactivity, lower general education than men, while men were more likely to smoke or drink alcohol. Women were less likely to be diagnosed with atherosclerotic cardiovascular disease (ASCVD) and heart failure but had higher frequency of kidney impairment (eGFR < 60 mL/min/1.73 m^2^) and any-site cancer than men. Men were 50–60% more likely to attain 3 or more treatment targets (defined as HbA_1c_ < 7%, blood pressure < 130/80 mmHg, risk-based LDL-cholesterol, and lack of central obesity) after adjusting for covariables. Care disparity was most evident in older women and in middle-income countries with infrequent access to trained educators and practice of self-monitoring of blood glucose.

**Implications of all the available evidence**

This is the first study with detailed comparison between men and women with T2D in Asia regarding their demographics, health habits, comorbidities, and control of risk factors. Women with T2D had worse quality of care than men especially in middle-income countries/areas, calling for targeted implementation programs to close these care gaps. Notably, we identified low physical activity levels and high proportions of central obesity in women. In men, we also identified unmet needs with suboptimal lifestyles including tobacco and alcohol consumption. These care disparities between high- and middle-income countries/areas imply considerable opportunities for improvement through enhancement of professional knowledge, patient education, care practices as well as health and social policies especially in middle-income countries.

## Introduction

Genetic, lifecourse, ecological, cultural, socioeconomical, cognitive-psychological-behavioral factors as well as access to care interact in an intertwined manner to influence the onset, trajectories, and outcomes in type 2 diabetes (T2D) and its complications.^1^ Apart from biological sex differences, social expectations and cultural norms may influence gender differences in quality of care and disease burden in different regions.^2^ These complexities can be further modified by differences in social policies, health systems, clinical practice, access to care, and self-management across different countries/areas.^1^^,^^3^

Given that half of the world population are women,^4^ recognition of possible gender differences in diagnosis, treatment, and control of T2D and associated risk factors is critically important for defining strategies to improve outcomes and reduce disease burden.^3^^,^^5^ Several reports indicated that women with T2D had a higher relative risk for major adverse cardiovascular events and heart failure than their male counterparts.^6^, ^7^, ^8^, ^9^ Even in high-income countries, women were less likely to receive guideline-recommended care and attain treatment targets for glycaemia and lipids,^5^^,^^10^ albeit similar data are lacking in Asia.

Although professional guidelines emphasize the use of a personalized approach to manage patients with T2D,^11^^,^^12^ there are few recommendations taking gender into consideration. This may reflect the paucity of observational and interventional data aimed at understanding gender-specific differences in care processes, perspectives, and unmet needs. Nearly 50% of adults with diabetes come from Asia,^4^ a region characterized by diversity in terms of ethnicities, cultures, and societies. There are few studies using structured data collection to compare gender differences in patients receiving care in different countries, areas, and cultures.^13^ Improved understanding of these gender-related disparities in health care, if any, can inform more precise and effective planning of health care delivery to reduce the burden of diabetes.

In a recent pooled analysis of 1 million Asian patients with diabetes, women were 2–3 times more likely to die from cardiovascular-renal events than men.^14^ We hypothesized that women with T2D were less likely to receive guideline-recommended care and attain treatment targets than men and tested this hypothesis by analysing data curated from a regional register established using the same protocol.

## Methods

### Study design – the Joint Asia Diabetes Evaluation (JADE) program

The rationale, design, and implementation of the JADE Program introduced in 2007 have been published.^15^ Briefly, the JADE Program entails the use of evidence-based, structured care protocols enabled by a web-based portal, allowing health care providers to establish their own registers for quality improvement with comparisons across countries/areas for benchmarking purpose (Supplementary Text). Adults aged ≥18 years with T2D who were either treated with oral/injectable glucose-lowering drugs or lifestyle modification were eligible.^16^ Patients with type 1 diabetes (defined as acute presentation with diabetic ketoacidosis, heavy ketonuria, or continuous insulin requirements within 1 year of diagnosis) were excluded.^16^ Data are subsequently pooled to establish an Asia register.

The web-based JADE portal incorporates templates for guiding workflow of structured evaluation complete with a validated risk engine, clinical decision support, and evidence-based care protocol.^15^ The JADE portal facilitates structured collection of clinical and biochemical data for risk stratification and prediction of 5-year cardiovascular-renal events and death.^15^ The nurses entered all anonymised data into the JADE portal followed by issuance and explanation of a personalised report to the patient to complement medical care. The JADE report summarizes the risk categories, probability of cardiovascular-renal events, and cardiometabolic risk factors with data visualization in the forms of colour codes, bar charts, and trend lines. It comprises tailored care recommendations for patients and health care providers. The JADE reports are available in eight languages (English, traditional Chinese, simplified Chinese, Indonesian, Korean, Malay, Thai, and Vietnamese).

The Chinese University Hong Kong (CUHK) Clinical Research Ethics Committee (CREC 2007.339) and the local ethics boards approved the implementation and evaluation of the JADE Program. All patients provided written informed consent. Participating sites were given access to the JADE portal designed and operated by the Asia Diabetes Foundation, a charitable research organization governed by the CUHK Foundation. All data were anonymized with each centre keeping their own patient log and having access to their own data for tracking and evaluation purpose.

### Data sources and study outcomes

Between 2007 and 2015, we registered 106,376 patients with T2D aged 18 years or older from participating sites in 11 countries/areas (China, Hong Kong, India, Indonesia, Korea, Malaysia, Philippines, Singapore, Taiwan, Thailand and Vietnam). Patients with T2D treated with diet with or without oral/injectable glucose-lowering drugs were eligible. We excluded patients with type 1 diabetes as previously defined.

Definitions of treatment targets were based on international recommendations during the data collection period, namely HbA_1c_ <7%,^17^ blood pressure (BP) <130/80 mmHg,^18^ risk-based low-density lipoprotein (LDL)-cholesterol (<2.6 mmol/L in high-risk patients or <1.8 mmol/L in very high-risk patients),^19^^,^^20^ and waist circumference (Asian cutoff points: <90 cm in men and <80 cm in women).^21^ Health habits in the last 3 months were self-reported and included adherence to balanced diet (yes, no, occasional, never), frequency of physical activity (≥30 min) per week (no regular physical activity, <3 times, 3–4 times, 5 times, >5 times), and self-monitoring blood glucose (SMBG) (<once monthly, ≥once monthly, ≥once weekly, ≥daily) (Supplementary Text). Atherosclerotic cardiovascular disease (ASCVD) was defined as a history of ischaemic heart disease, myocardial infarction, coronary artery intervention, stroke, or peripheral arterial disease (lower-extremity amputation, peripheral revascularisation, or an ankle-brachial index <0.9).^16^^,^^22^

### Statistical analysis

All patients with T2D who fulfilled eligibility criteria and were registered in the JADE Program between 2007 and 2015 were analysed. Data are presented as mean ± standard deviation (SD) or median (interquartile range [IQR]) for continuous variables with normal or skewed distribution, respectively. Categorical variables are presented as number and percentages. For two-group comparison of continuous variables, we used independent *t*-test for data with normal distribution and Wilcoxon rank-sum test for other data. We used Chi-square test for between-group comparisons of categorical variables.

We converted coded responses of health habits data into binary variables for the purpose of analysis. To examine for gender differences in attaining ≥3 treatment targets, we performed multiple logistic regression analysis using gender as the dependent variable, adjusting for countries and centres as random intercept with centres nested in countries, along with further adjustment for year of registration as a linear continuous variable. In model 1, we included age at registration, duration of diabetes, college education, and year of registration. In Model 2, additional variables included current smoker, regular alcohol drinker, adherence to balanced diet, physical activity ≥3 times/week, and SMBG ≥once/week. In Model 3, additional variables included prior ASCVD, heart failure, any-site cancer, and estimated glomerular filtration rate (eGFR) <60 mL/min/1.73 m^2^. Model 4 included additional variables of use of oral glucose-lowering drugs, insulin, blood pressure-lowering drugs, and statin.

We performed subgroup analyses stratified by age of natural menopause (<50 years *vs.* ≥50 years),^23^ prior ASCVD/heart failure, eGFR <60 mL/min/1.73 m^2^, and national income level, namely high-income (Hong Kong, Taiwan, Singapore, and Korea) and middle-income countries/areas (China, Vietnam, Thailand, Indonesia, India, Malaysia, and Philippines),^24^ as appropriate. We performed pairwise deletion for variables with missing values as well as multiple imputations. We used multivariate imputation by chained equations with 20 imputations to handle missing data.^25^ The analysis after imputations were combined by the Rubin rule.

All analyses were conducted using R 4.2.1 (https://www.r-project.org/). Given that there were no multiple comparisons, no adjustment for multiple testing was required. A 2-tailed p-value <0.05 was considered statistically significant.

### Role of the funding source

The funder of the study, Asia Diabetes Foundation, designed, administered, and implemented the JADE Program with data collection, data analysis, data interpretation, and writing of the report. The corresponding author had full access to all data in the study and had the final responsibility for the decision to submit for publication.

## Results

**Fig. 1:**
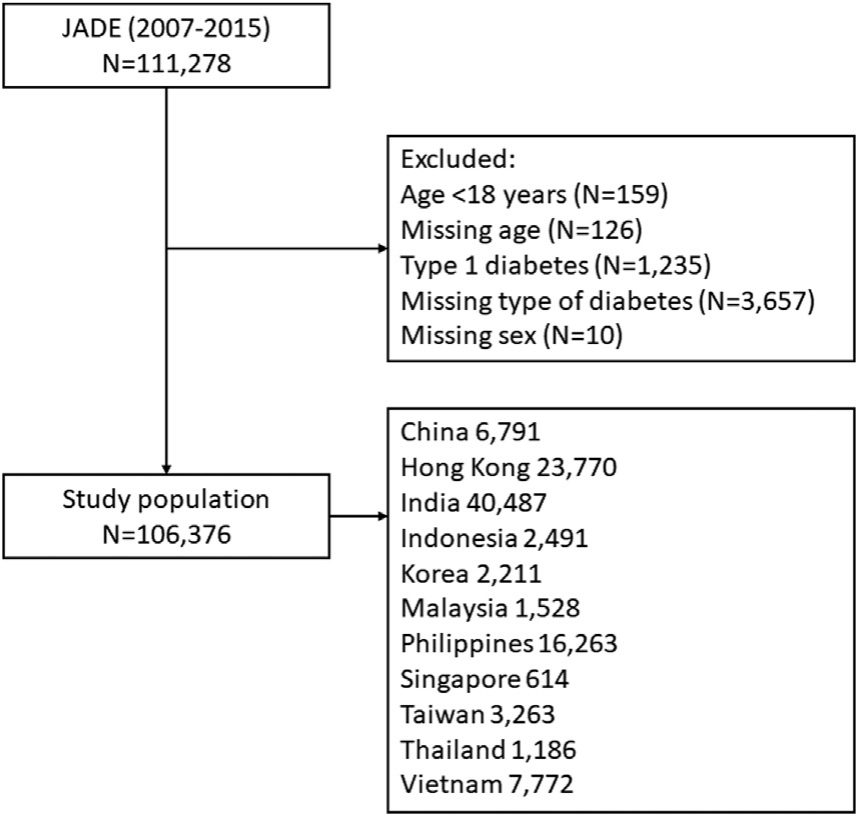
Study flow.

A total of 106,376 patients with T2D (2007–2015) were included in the present analysis (Fig. 1), of whom 56,552 (53.2%) were men. Supplementary Table S1 shows the number of patients by countries/areas and year of registration.

Table 1 compares the clinical characteristics by gender. The mean ± SD HbA_1c_ of the entire cohort was 8.0 ± 1.9% and one-third of them attained HbA_1c_ <7% at baseline. Compared with men, women were older (mean ± SD age: 58.1 ± 11.9 *vs.* 55.9 ± 11.9 years), had lower eGFR (78.5 ± 24.8 *vs.* 82.0 ± 24.0 mL/min/1.73 m^2^) and were less likely to receive college education (28.9% *vs.* 48.8%). Compared with men, women were more likely to attain BP <130/80 mmHg (29.4% *vs.* 25.7%), but not for risk-based LDL-cholesterol target (16.2% *vs.* 18.8%). Despite having a lower proportion of general obesity (BMI < 25 kg/m^2^; 54.8% *vs.* 57.8%), women were more likely to have central obesity than men (77.5% *vs.* 57.3%). After adjusting for covariables, men had an odds ratio of 1.52–1.63 for the attainment of 3 or more treatment targets (defined as HbA_1c_ < 7%, blood pressure < 130/80 mmHg, risk-based LDL-cholesterol, and lack of central obesity) than women (Table 2). Women were less likely to smoke or drink alcohol, perform regular physical activity (41.2% *vs.* 48.2%), or SMBG (34.0% *vs.* 35.6%). Fewer women had exposure to allied health personnel (nurse educators: 51.8% *vs.* 54.9%; dietitians: 59.0% *vs.* 63.7%; podiatrists: 29.1% *vs.* 32.9%) than men. Women were less likely to be diagnosed with ASCVD (12.8% *vs.* 17.0%) and heart failure (1.3% *vs.* 2.3%) but more likely to have kidney impairment (eGFR < 60 mL/min/1.73 m^2^: 22.3% *vs.* 17.6%) and any-site cancer events (2.5% *vs.* 1.6%) than men. Similar patterns were observed when analysed by national income level (Supplementary Table S2 and S3).

**Table 1:** Cardiovascular risk profiles and quality of care among patients with type 2 diabetes in Asia (2007–2015).

	Women (n = 49,824)		Men (n = 56,552)	
	n		n	
Country/Area, n (%)	49,824		56,552	
China		2984 (6.0%)		3807 (6.7%)
Hong Kong		10,648 (21.4%)		13,122 (23.2%)
India		16,520 (33.2%)		23,967 (42.4%)
Indonesia		1180 (2.4%)		1311 (2.3%)
Korea		1007 (2.0%)		1204 (2.1%)
Malaysia		732 (1.5%)		796 (1.4%)
Philippines		9649 (19.4%)		6614 (11.7%)
Singapore		319 (0.6%)		295 (0.5%)
Taiwan		1478 (3.0%)		1785 (3.2%)
Thailand		796 (1.6%)		390 (0.7%)
Vietnam		4511 (9.1%)		3261 (5.8%)
College education, n (%)	44,485	12,838 (28.9%)	51,260	25,016 (48.8%)
Family history of diabetes, n (%)	44,446	26,180 (58.9%)	50,897	29,867 (58.7%)
Age, years	49,750	58.1 ± 11.9	56,500	55.9 ± 11.9
Duration of diabetes^a^, years	47,236	6.0 (2.0–12.0)	53,856	6.0 (2.0–12.0)
Body mass index, kg/m^2^	45,298	26.3 ± 4.9	52,707	26.1 ± 4.2
Waist circumference, cm	37,676	88.4 ± 12.3	45,779	92.1 ± 11.2
HbA1c, %	40,806	8.0 ± 1.9	48,285	8.0 ± 1.9
Systolic blood pressure, mmHg	47,631	131.0 ± 17.7	54,553	131.2 ± 16.8
Diastolic blood pressure, mmHg	47,541	78.0 ± 9.5	54,441	79.9 ± 9.4
Total cholesterol, mmol/L	38,576	4.7 (4.0, 5.4)	44,745	4.5 (3.9, 5.2)
Triglyceride^a^, mmol/L	39,870	1.5 (1.1–2.1)	47,507	1.6 (1.1–2.2)
HDL-cholesterol, mmol/L	39,184	1.2 (1.0, 1.4)	46,463	1.1 (0.9, 1.3)
LDL-cholesterol, mmol/L	38,950	2.6 (2.1, 3.3)	46,162	2.5 (2.0, 3.1)
Non-HDL cholesterol, mmol/L	37,182	3.4 (2.7, 4.1)	43,019	3.3 (2.7, 4.0)
eGFR, mL/min/1.73 m^2^	37,783	78.5 ± 24.8	45,435	82.0 ± 24.0
Cardiometabolic risk factors, n (%)				
Very high CVD risk^b^	47,737	47,232 (98.9%)	54,795	54,288 (99.1%)
HbA_1c_ < 7%	40,806	13,940 (34.2%)	48,285	16,298 (33.8%)
Blood pressure < 130/80 mmHg	47,559	14,004 (29.4%)	54,458	14,005 (25.7%)
Attained risk-based LDL-cholesterol target^d^	38,370	6292 (16.2%)	45,934	8627 (18.8%)
General obesity (BMI ≥ 25 kg/m^2^)	45,298	24,807 (54.8%)	52,707	30,447 (57.8%)
Central obesity (waist circumference ≥90 cm in men or ≥80 cm in women)	37,676	29,184 (77.5%)	45,779	26,251 (57.3%)
≥3 treatment targets attained^c^	31,388	2394 (7.6%)	38,277	4046 (10.6%)
Current smoker	47,703	800 (1.7%)	54,734	11,443 (20.9%)
Regular alcohol drinker	47,645	165 (0.3%)	54,596	4501 (8.2%)
Self-reported health habits in the last 3 months, n (%)				
Adherence to balanced diet	46,590	39,207 (84.2%)	52,692	43,296 (82.2%)
Physical activity ≥3 times/week	47,201	19,427 (41.2%)	53,548	25,803 (48.2%)
SMBG ≥ once/week	42,330	14,380 (34.0%)	47,644	16,983 (35.6%)
Ever exposure to allied health professionals, n (%)				
Education by nurses	46,751	24,199 (51.8%)	52,851	29,006 (54.9%)
Education by dietitians	47,417	27,975 (59.0%)	53,915	34,369 (63.7%)
Education by podiatrists	44,860	13,068 (29.1%)	50,208	16,523 (32.9%)
Comorbidities, n (%)				
ASCVD	49,824	6389 (12.8%)	56,552	9621 (17.0%)
Heart failure	49,824	641 (1.3%)	56,552	1283 (2.3%)
eGFR < 60 mL/min/1.73 m^2^	37,783	8443 (22.3%)	45,435	8006 (17.6%)
Any-site cancer	49,824	1225 (2.5%)	56,552	921 (1.6%)
Medication use, n (%)				
Oral glucose-lowering drugs	49,824	42,644 (85.6%)	56,552	48,421 (85.6%)
Injectable GLP1-RA	49,824	105 (0.2%)	56,552	145 (0.3%)
Insulin	49,824	13,459 (27.0%)	56,552	14,987 (26.5%)
Blood pressure-lowering drugs	49,824	29,251 (58.7%)	56,552	31,778 (56.2%)
Renin-angiotensin system inhibitors	42,123	15,954 (37.9%)	48,661	18,472 (38.0%)
Statin	42,058	16,963 (40.3%)	47,926	19,518 (40.7%)
Aspirin	49,824	6350 (12.7%)	56,552	9315 (16.5%)

Footnotes: Data are presented as mean ± standard deviation.

ASCVD, atherosclerotic cardiovascular disease; BMI, body mass index; eGFR, estimated glomerular filtration rate; GLP1-RA, glucagon-like peptide 1 receptor analogues; HDL, high-density lipoprotein; LDL, low-density lipoprotein; SMBG, self-monitoring blood glucose.

a Median (interquartile range) or number (percentage).

b Definition of risk for cardiovascular disease was based on the 2016 European Society of Cardiology/European Atherosclerosis Society (ESC/EAS) recommendations in line with the data collection period.

c We defined ≥3 treatment targets attained as 1) HbA_1c_ <7%, 2) blood pressure <130/80 mmHg.

d 3) Risk-based LDL-cholesterol target (<2.6 mmol/L if high risk or <1.8 mmol/L if very high-risk), and 4) lack of central obesity (waist circumference <90 cm in men or <80 cm in women).

**Table 2:** Logistic regression analysis for attainment of 3 or more treatment targets in men, compared with women.

	Complete data		Imputed data	
	OR (95% CI)	p-value	OR (95% CI)	p-value
Model 1	1.47 (1.38, 1.56)	<0.001	1.38 (1.31, 1.45)	<0.001
Model 2	1.61 (1.51, 1.71)	<0.001	1.50 (1.42, 1.58)	<0.001
Model 3	1.63 (1.53, 1.75)	<0.001	1.52 (1.44, 1.60)	<0.001
Model 4	1.63 (1.51, 1.74)	<0.001	1.52 (1.44, 1.60)	<0.001

Footnotes: Random intercept of countries/areas and centres nested in countries/areas.

Model 1: age at registration, duration of diabetes, college education, and year of registration.

Model 2: Model 1 + current smoker, regular alcohol drinker, adherence to balanced diet, physical activity ≥3 times/week, and SMBG ≥once/week.

Model 3: Model 2 + prior ASCVD, heart failure, any-site cancer, and eGFR <60 mL/min/1.73 m^2^.

Model 4: Model 3 + use of oral glucose-lowering drugs, insulin, blood pressure-lowering drugs, and statin.

**Fig. 2:**
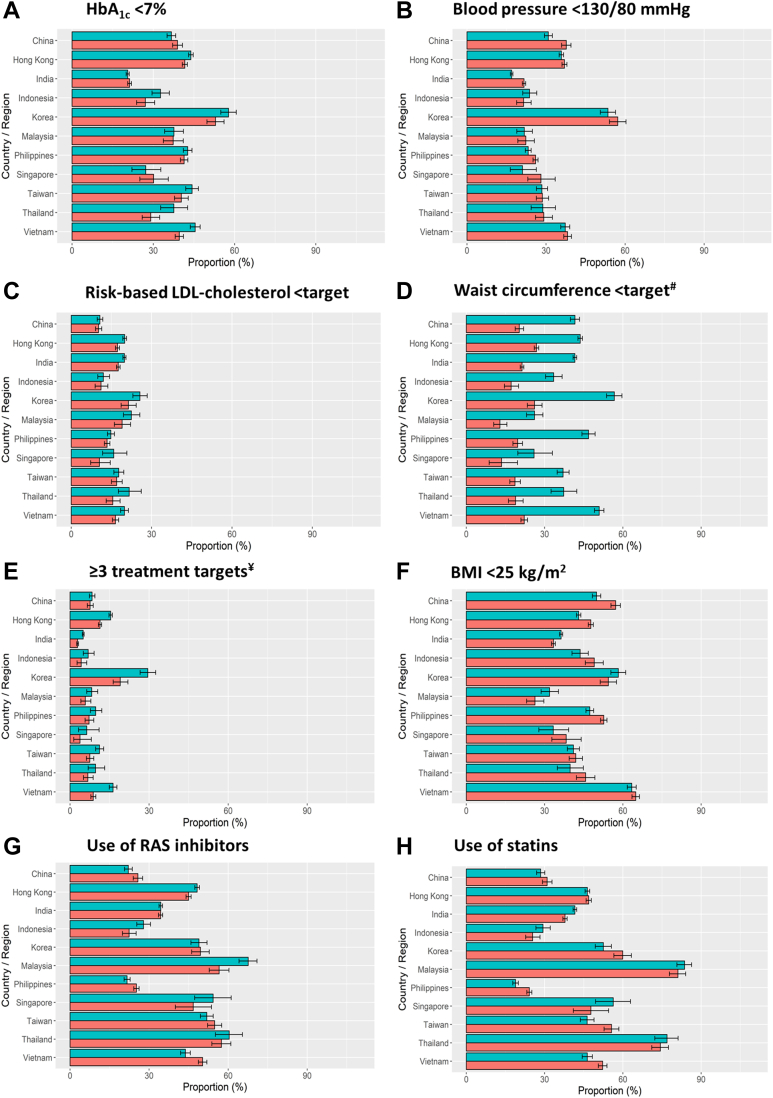
Control of cardiometabolic risk factors (HbA1c < 7% (A), blood pressure < 130/80 mmHg (B), risk-based LDL-cholesterol target (C), waist circumference < target (D), ≥3 treatment targets (E) and BMI < 25 kg/m^2^ (F)) and use of cardiorenal protective drugs (use of RAS inhibitors (G) and use of statins (H)) at registration by countries/areas. Footnotes: Data are available in Supplementary Tables S4–S11. Green bars represent men. Orange bars represent women. ^γ^Definition of CV risk was based on the 2016 European Society of Cardiology/European Atherosclerosis Society (ESC/EAS) recommendations in line with the data collection period. ^#^Treatment targets were defined as HbA_1c_ <7%, blood pressure <130/80 mmHg, risk-based LDL-cholesterol target (<2.6 mmol/L if high risk or <1.8 mmol/L if very high-risk), and lack of central obesity (waist circumference <90 cm in men or <80 cm in women). General obesity was defined as body mass index ≥25 kg/m^2^. RAS, renin-angiotensin system.

Fig. 2 and Supplementary Tables S4–S11 show the gender differences in the control of cardiometabolic risk factors and use of cardiorenal protective drugs across 11 countries/areas. Korea had the highest proportion of patients attaining HbA_1c_ <7% and BP <130/80 mmHg, whilst the lowest proportion was in India for both indicators (Fig. 2A and B). Notwithstanding minor differences amongst countries, overall, men were more likely to attain HbA_1c_ <7% than women. Fewer than one-third of the entire cohort attained risk-based LDL-cholesterol target with a lower proportion in women (Fig. 2C). In all countries/areas, women were more likely to have central obesity (Fig. 2D), despite having a lower proportion with BMI <25 kg/m^2^ (Fig. 2F). In Korea, 30% of patients attained ≥3 treatment targets *vs.* 10–20% in other countries (Fig. 2E). More men attained ≥3 treatment targets than women by countries/areas except China and Philippines which did not show gender differences (Fig. 2E). More than 40% of patients received statins or RAS inhibitors with lower proportions in China, India, Indonesia, and Philippines (20%–35%) (Fig. 2G and H).

**Fig. 3:**
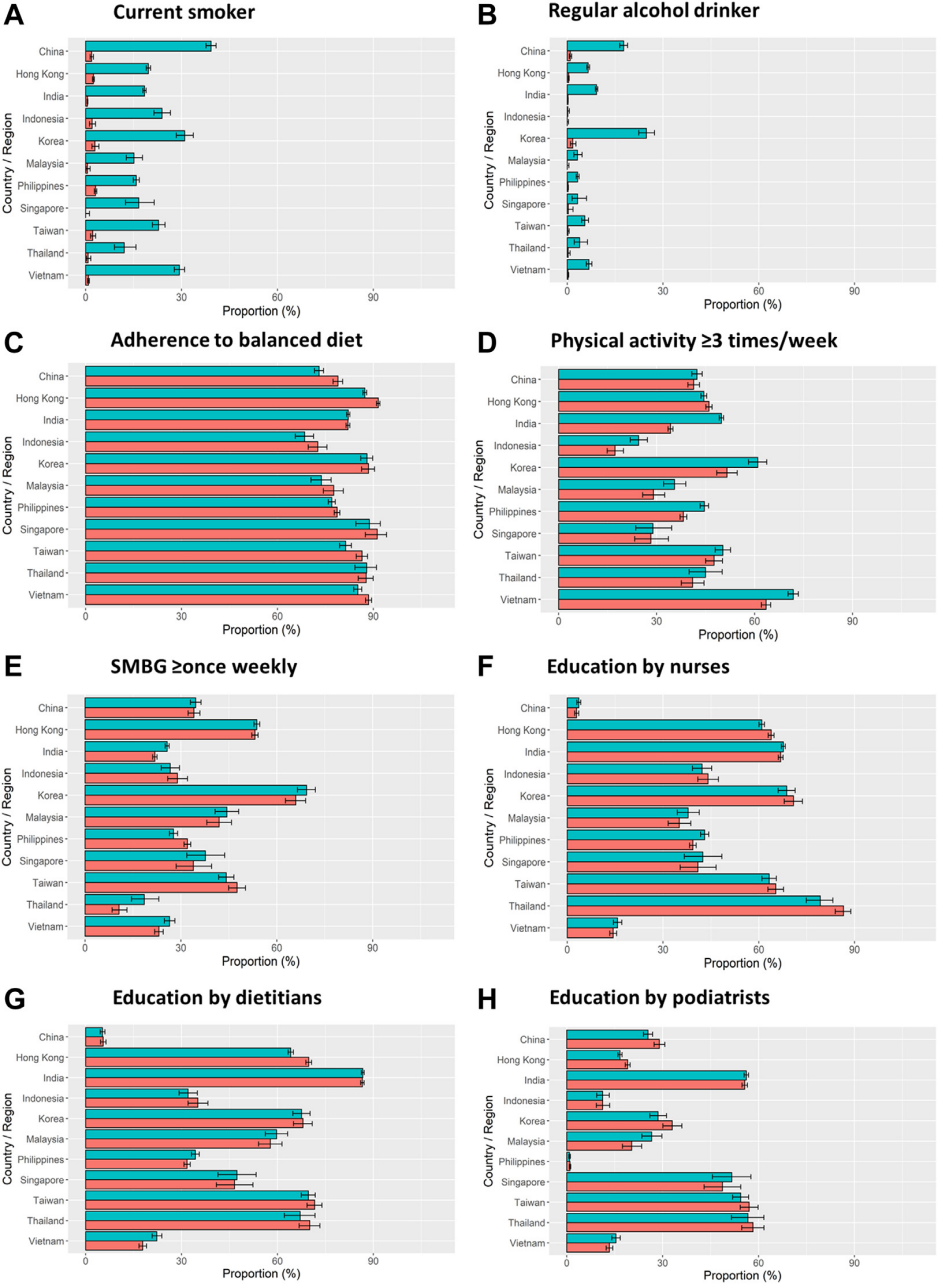
Self-reported health habits in the past three months (current smoker (A), regular alcohol drinker (B), adherence to balanced diet (C), physical activity ≥3 times/week (D) and SMBG ≥ once weekly (E)) and exposure to educators (education by nurses (F), education by dietitians (G) and education by podiatrists (H)) at registration by countries/areas. Footnotes: Data are available in Supplementary Tables S12–S19. Green bars represent men. Orange bars represent women. SMBG, self-monitoring blood glucose.

**Fig. 4:**
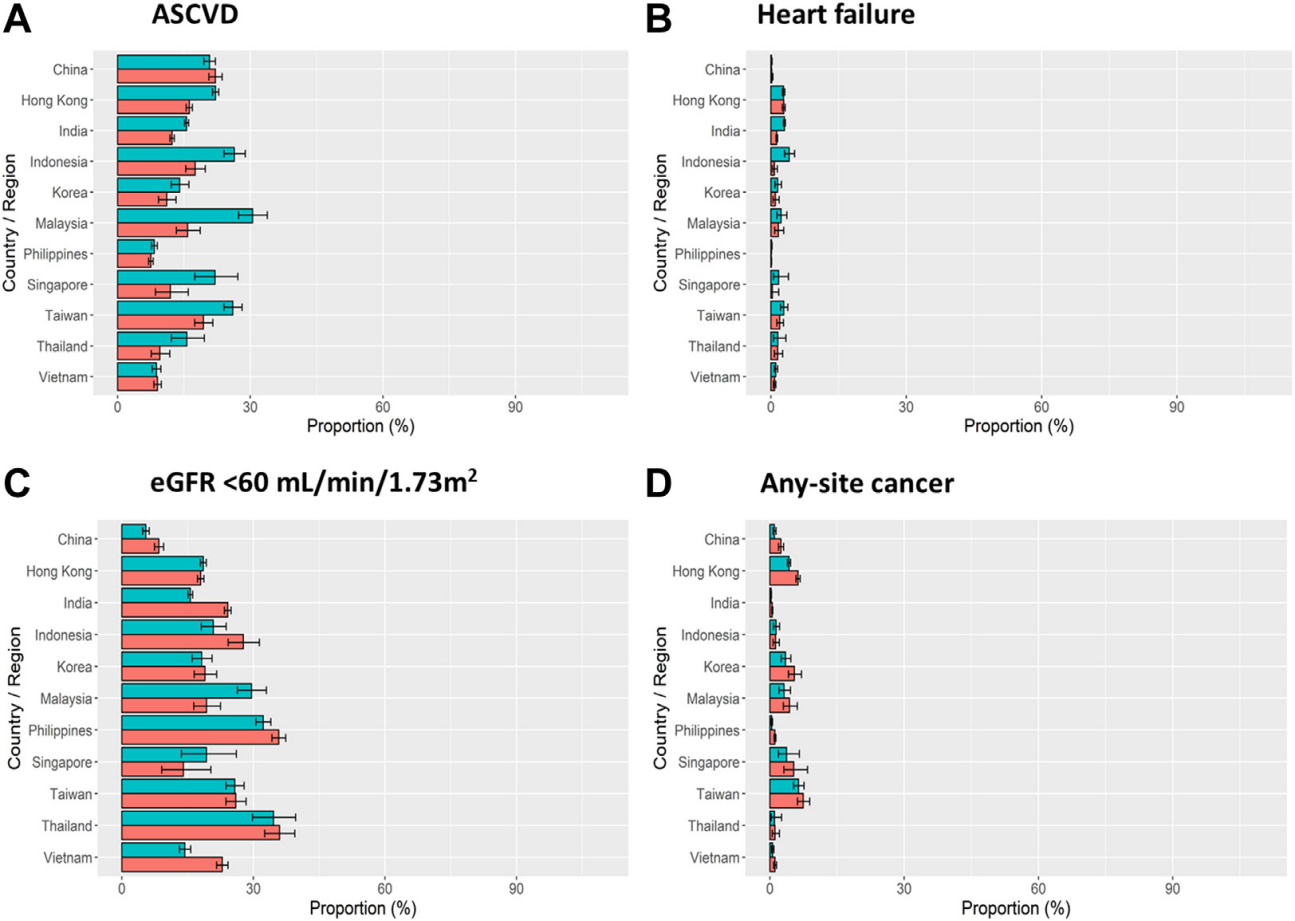
Diabetes-related comorbidities (ASCVD (A), heart failure (B), eGFR < 60 mL/min/1.73 m^2^ (C) and any-site cancer (D)) at registration by countries/areas. Footnotes: Data are available in Supplementary Tables S20–S23. Green bars represent men. Orange bars represent women. ASCVD, atherosclerotic cardiovascular disease; eGFR, estimated glomerular filtration rate (creatinine-based CKD-EPI formula).

Fig. 3 and Supplementary Tables S12–S19 show country/area-specific and gender differences in self-reported health habits and referral to patient education by allied health personnel. In both high- and low-income countries/areas, men were more likely to smoke (Fig. 3A) and drink alcohol (Fig. 3B). In high-income countries/areas, more than 70% of the cohort reported adherence to balanced diet (Fig. 3C). In middle-income countries/areas, men were more likely to exercise ≥3 times per week than women (Fig. 3D and E). Fig. 4 and Supplementary Tables S20–S23 compare the proportion of patients with comorbidities by countries/areas and gender with the highest proportion of ASCVD in Malaysia. Men were more likely to be diagnosed with ASCVD (Fig. 4A) and heart failure (Fig. 4B) than women who were more likely to have eGFR <60 mL/min/1.73 m^2^ notably in China, India, Indonesia, Philippines, Thailand, and Vietnam (Fig. 4C). In all countries/areas, women were more likely to have any-site cancer than men (Fig. 4D).

Women aged ≥50 years defined as menopausal age, were more likely to have ASCVD, heart failure, eGFR <60 mL/min/1.73 m^2^, and any-site cancer than young women. They were more likely to be treated for cardiometabolic risk factors but less likely to attain treatment targets than younger women (Supplementary Table S24). Compared with men aged ≥50 years, older women were more likely to adhere to balanced diet and not to smoke or drink alcohol but less likely to perform regular exercise. They were less likely to attain risk-based LDL-cholesterol target and receive education from nurses, dietitians, and podiatrists with lower frequency of SMBG than men (Supplementary Tables S24 and S25 and Supplementary Fig. S1A). Among patients with prior ASCVD and/or heart failure, women had lower usage of RAS inhibitors or statins than men (Supplementary Fig. S1B and Supplementary Table S26). Among the four treatment targets, only 20.9%, 21.1%, 24.0%, and 30.6% of women attained risk-based LDL-cholesterol target, waist circumference <80 cm, blood pressure <130/80 mmHg, and HbA_1c_ <7%, respectively (Supplementary Fig. S1B and Supplementary Table S26). The corresponding attainment rates were 24.1%, 41.2%, 23.7%, and 33.4% in men (Supplementary Fig. S1B and Supplementary Table S26). Among patients with eGFR <60 mL/min/1.73 m^2^, use of statins and RAS inhibitors were less frequent in women than in men (Supplementary Fig. S1C and Supplementary Table S27). Although both men and women with comorbidities reported >80% adherence to balanced diet, fewer than 50% performed SMBG and exercise ≥3 times per week especially in women (Supplementary Fig. S1B and C, and Supplementary Tables S26 and S27). Compared with patients who were analysed, men and women excluded had worse risk factor control and less likely to report having comorbidities, practice good health habits, and receive education from allied health personnel and organ-protective medications (Supplementary Tables S28 and S29).

## Discussion

In this cross-sectional analysis of the prospective JADE cohort involving 11 countries/areas in Asia, we highlighted several gender differences in cardiometabolic risk profiles, self-reported health behaviours and quality of care. Compared with men, women were more likely to have central obesity, kidney impairment, any-site cancer but less likely to be diagnosed with ASCVD and heart failure than men. Women were less likely to attain multiple treatment targets than men and amongst patients with cardiorenal disease, women were less likely to receive statins and RAS inhibitors. From a self-management perspective, use of tobacco and alcohol was uncommon in women but they were less likely to perform regular exercise, receive education from allied health personnel, or perform SMBG. This gender disparity might partly contribute to the 2–3 times higher risk of death due to cardiovascular-renal diseases in women compared to men in Asia.^14^ Similarly, the care disparity between high- and middle-income countries/areas in our analysis concord with the 2 times higher death rate due to coronary artery disease in India compared to UK and USA in the Million Death Study.^26^ Taken together, these findings highlighted the gender differences in education level, health habits, self-management, risk factors, comorbidities, and quality of care especially in middle-income countries/areas undergoing socioeconomical transition.

The overall control of hypertension was higher in women than in men although less so in those aged ≥50 years. This is likely due to the lower BMI in women which is a major determinant for BP.^27^ In the population-based Korea National Health and Nutrition Examination Survey, women also had lower BP than men.^28^ According to the UK Prospective Diabetes Study, among the three risk factors of BP, glycaemia, and lipids, control of BP had the largest effect size in reducing cardiovascular events.^29^ Apart from BMI,^27^ salt or sodium intake are important determinants of BP.^30^ The slow progress in national salt reduction programs^30^ and suboptimal control of BP amongst treated patients, call for accelerated implementation, monitoring, and treatment strategies to improve control of hypertension in Asia.

In the overall cohort, we did not observe gender difference in either HbA_1c_ <7% attainment or practice of SMBG although the latter was less frequent in middle-income than high-income countries/areas. There are many system- and patient-level barriers in promoting SMBG.^31^ These included customs and import duties, reimbursement policies, and out-of-pocket payment with considerable variations between low- and middle-income countries/areas as well as across countries with similar income levels.^31^^,^^32^ In the International Diabetes Federation Life for a Child Program involving patients with type 1 diabetes from 37 low- and middle-income countries, blood glucose test strips were provided by payors only in 14% of the low-resourced health care systems. In T2D, reimbursement for blood glucose test strips was uncommon in many countries.^33^ Effective use of SMBG required good health literacy and numeracy.^31^ In many low- and middle-income countries in Asia, low education attainment in women might limit employment opportunity and earning capacity to pay for health care including blood glucose test strips. In this study, only 28.9% of women received college education compared with 48.8% in men. Lack of availability and accessibility to diabetes education and support program essential for teaching patients how to perform SMBG, interpret results, and adjust dietary patterns or therapy including insulin titration is a challenge in low-income countries as well as low-resource settings in high-income countries/areas.^1^^,^^32^^,^^34^ Interestingly, middle-income countries/areas had lower proportions of patients exposed to nurses and dietitians but higher proportions to podiatrists than high-income countries/areas, suggesting there might be a higher service needs for foot problems in these countries.

Men and women had different biomedical-psychosocial-behavioral determinants in disease initiation and progression.^3^^,^^35^ In line with other epidemiological studies, we noted that women, irrespective of their status of menopause, were more likely to have central obesity than men.^36^, ^37^, ^38^ This is especially true among postmenopausal women who experienced body fat redistribution with increased visceral adiposity.^3^^,^^5^^,^^39^ Changes in hormonal profiles, negative emotions, physical inactivity, and insufficient ongoing support for self-management might worsen these gender differences.^40^, ^41^, ^42^ In high-income countries, social disparity was associated with high prevalence of overweight or obesity in both gender with larger effect size in women.^5^ In our study, the low level of education might contribute to the higher frequency of central obesity in women.

The male predominant nature in cardiovascular-kidney outcome trials might contribute to the perceived low risk for these complications among health care providers and patients with delayed screening, diagnosis, and intervention in women. In population-based surveys, 4.0% and 1.9% of adult respondents in Malaysia and Thailand, respectively, were aware of the diagnosis of kidney disease.^43^^,^^44^ In the prospective Asia Cohort Consortium, women had 2–3 times higher risk of death due to cardiorenal diseases, especially among those aged <50 years at baseline.^14^ Similar to many cross-sectional analysis, women in our study were less likely to be diagnosed with ASCVD and heart failure than men. Among those diagnosed with ASCVD and/or heart failure, women were also less likely to receive cardiorenal protective drugs with worse control of cardiometabolic risk factors than men. In keeping with their high risk for cardiorenal death,^14^ women in our analysis also had a higher prevalence of kidney impairment than men. The biological mechanisms for sex differences in kidney diseases in patients with T2D were poorly understood.^45^ However, both epidemiological and interventional studies supported a causal association between obesity and kidney diseases.^46^, ^47^, ^48^ To this end, women were more likely to have central obesity than men in our analysis.

Although we did not assess psychological health in this cohort, women with T2D are known to have higher prevalence of depression and distress than men.^42^^,^^49^ These emotional factors can activate neurohormonal system with increased visceral obesity and cardiometabolic risk.^1^^,^^42^ In Singapore, cluster analysis of health care data indicated that young women with depression and old women with comorbidities including depression had the largest health care utilization.^50^ Of note, there is huge data gap in patient-reported outcomes which are important determinants for health behavior, self-management, and clinical outcomes in T2D.^1^ Our real-world evidence should alert health care providers to assess cardiorenal risk and symptoms in women and intensify control of cardiometabolic risk factors including use of cardiorenal protective drugs to reduce this gender care disparity.^51^^,^^52^

The major strength of our study is the shared protocol for structured data collection which allows comparisons across a large cohort of patients with T2D from diverse clinic settings in 11 countries/areas in Asia. The International Diabetes Management Practices Study (IDMPS), another large real-world register, also examined the pattern of care practices outside the USA and Western Europe.^53^ Despite the increasing number of innovative medications and technologies, the attainment rate of HbA_1c_ <7% remained <40% in 2005–2017.^53^ However, the question of gender disparities was not examined in the IDMPS. Second, in the JADE Program, comorbidities were recorded by attending physicians and trained allied health personnel based on direct history taking with verification from medical records. The JADE Program is one of the largest quality improvement programs which has provided new insights regarding unmet needs in patients with T2D in Asia. In a recent report analysing nation- or territory-wide data from 16 high-income countries/areas, East Asian countries/areas including Hong Kong, Singapore, South Korea, and Taiwan had the largest decrement in annual death rates of 3%–4% per year in 1995–2016.^54^ The large number of publications on registers and quality improvement programs from Asia during the last two decades might have contributed to multi-stakeholder efforts to reform health care delivery and improve care standards through many measures not limited to universal health coverage and implementation of diabetes risk assessment and education programs.^16^^,^^22^

There are also study limitations. First, due to the pragmatic nature of the program, we could not exclude volunteer or recruitment bias due to enthusiasm of patients and health care providers. Patients who were severely ill, older than 80 years, socially disadvantaged with restricted access to care or suboptimal control of cardiometabolic risk factors, might not be represented, biasing our findings to those with less severe disease. Second, factors such as higher illness burden might influence one's motivation to participate in the JADE Program and willingness to pay out-of-pocket for medical care. This might bias our findings to patients with more severe disease. That said, our results are consistent after adjustment for countries and centres/clinics attended by different health care providers (primary care *vs.* specialist care) in different care systems (fully/partially subsidized *vs.* private), making our conclusions generalisable. Third, we acknowledged potential bias with self-reported health habits which could be influenced by education and cultural background. However, these socioeconomical and health habit data are often captured in real-world practice for clinical management with prognostic significance.^55^ Due to the pragmatic nature of the register, we limited the number of data collected in these non-experimental settings. As we gathered more evidence on the utility of patient-reported outcomes (e.g., emotional well-being) for prognostication and intervention purposes, these variables will be included in these structured assessments in the future. Last, given that site participation in the JADE Program was entirely voluntary with no reimbursement to the sites, patients, or health care providers, this register-based analysis was not intended to represent the situation of a country/area, but mainly used to identify care gaps and perform risk analysis. The JADE portal was designed as a tool to facilitate management and gather data to inform practice. To this end, the variations between and within country highlight the enormous opportunities for change.

In conclusion, in this large multinational cohort with T2D in Asia, there were gender differences in health habits, cardiometabolic risk factors, and comorbidities with women being less likely to be physically active, perform SMBG, and receive cardiorenal protective drugs and education from nurses/dietitians accompanied by reduced likelihood of attaining treatment targets (including central obesity). This disparity is particularly evident in older women and in middle-income countries/areas. Given the increased risk of cardiovascular-renal death in women with T2D in Asia, our results call for more focused efforts to understand and manage the biomedical-psychosocial-behavioural needs especially in older women and in middle-income countries/areas to reduce this care gap.

## Contributors

J.C.N.C. conceptualized the work. All authors and collaborators were involved in the patient recruitment. E.S.H.L. and L.-L.L. performed the analysis with support from J.C.N.C. L.-L.L. wrote the first draft and J.C.N.C. finalized the manuscript. All authors participated in the research methodology, data interpretation, manuscript revision for important intellectual content, and approved the final version of the manuscript. The corresponding author attests that all listed authors meet authorship criteria and that no others meeting the criteria have been omitted.

## Data sharing statement

Data cannot be shared publicly as we did not have patients’ consent to release the data in the public domain for open, unrestricted access. Researchers who are interested and meet the criteria for research access to our data may apply via Asia Diabetes Foundation (enquiry@adf.org.hk).

## Declaration of interests

J.C.N.C. is the Chief Executive Officer of ADF on a pro-bono basis. J.C.N.C. reported receiving grants through her affiliated institutions and/or honoraria for consultancy or giving lectures from AstraZeneca, Bayer, Boehringer Ingelheim, Celltrion, Eli Lilly, Hua Medicine, Lee Powder, Merck, Merck Sharp & Dohme, Novartis, Novo Nordisk, Pfizer, Sanofi, Servier, and Viatris Pharmaceutical. R.C.W.M. reported receiving grants and/or honoraria for consultancy or giving lectures from AstraZeneca, Bayer, Boehringer Ingelheim, Eli Lilly, Pfizer, and Takeda. A.O.Y.L. reported receiving grants through her affiliated institutions and/or honoraria for consultancy and/or giving lectures and/or travelling support from Amgen, AstraZeneca, Bayer, Biogen, Boehringer Ingelheim, Eli Lilly, Hospital Authority, Lee Pharmaceutical, MSD, Novo Nordisk, Roche, Sanofi. A.O.Y.L. is the Deputy Chair of the Joint-Chinese University of Hong Kong – New Territories East Cluster Clinical Research Ethics Committee Phase 1 Panel, Deputy Director of Clinical Research Management Office of the Chinese University of Hong Kong and Vice President of the Hong Kong Association on the Study of Obesity. L.-L.L. reported receiving grants through her affiliated institutions and/or honoraria for consultancy and giving lectures from Abbott, AstraZeneca, Boehringer Ingelheim, Merck Sharp & Dohme, Novo Nordisk, Pfizer, Roche, Sanofi, Servier, and Zuellig Pharma. T.K.N. reported receiving honoraria for consultancy and/or giving lectures and/or travelling support from Boehringer Ingelheim and Servier. S.-Y.G. department received payment for lectures/presentations/speakers bureaus/manuscript writing/educational events from Boehringer Ingelheim, Bayer, and Medtronic, unrelated to this manuscript. Other authors declared no potential conflict of interest.
