# Supplementary material for: Gender-associated cardiometabolic risk profiles and health behaviors in patients with type 2 diabetes: a cross-sectional analysis of the Joint Asia Diabetes Evaluation (JADE) program
Source: Lancet Reg Health West Pac. 2022 Dec 19;32:100663. doi: 10.1016/j.lanwpc.2022.100663 (PMC9918795; doi:10.1016/j.lanwpc.2022.100663)
Supplement: Supplementary Text, Tables S1–S29 and Figure S1 [file mmc1.docx]

**Cardiometabolic Risk Profiles and Health Behaviors in Patients with Type 2 Diabetes By Gender: A Cross-Sectional Analysis of The Joint Asia Diabetes Evaluation (JADE) Program**

**Appendix**

Table of Contents

[Supplementary Text. A summary description of the JADE Program^1^ 3](#_Toc118150038)

[Supplementary Table 1. Number of patients by countries/areas and year of registration to the JADE Program 7](#_Toc118150039)

[Supplementary Table 2. Cardiovascular risk profiles and quality of care among patients with type 2 diabetes from middle-income countries/areas in Asia (China, India, Indonesia, Malaysia, Philippines, Thailand, and Vietnam) 8](#_Toc118150040)

[Supplementary Table 3. Cardiovascular risk profiles and quality of care among patients with type 2 diabetes from high-income countries/areas in Asia (Hong Kong SAR, Singapore, South Korea, and Taiwan) 10](#_Toc118150041)

[Supplementary Table 4. Data table for HbA_1c_ <7% at registration by countries/areas (Figure 2A in the main text) 12](#_Toc118150042)

[Supplementary Table 5. Data table for blood pressure <130/80 mmHg at registration by countries/areas (Figure 2B in the main text) 13](#_Toc118150043)

[Supplementary Table 6. Data table for risk-based LDL-cholesterol target at registration by countries/areas (Figure 2C in the main text) 14](#_Toc118150044)

[Supplementary Table 7. Data table for waist circumference below target at registration by countries/areas (Figure 2D in the main text) 15](#_Toc118150045)

[Supplementary Table 8. Data table for ≥3 treatment targets at registration by countries/areas (Figure 2E in the main text) 16](#_Toc118150046)

[Supplementary Table 9. Data table for BMI <25 kg/m^2^ at registration by countries/areas (Figure 2F in the main text) 17](#_Toc118150047)

[Supplementary Table 10. Data table for use of RAS inhibitors at registration by countries/areas (Figure 2G in the main text) 18](#_Toc118150048)

[Supplementary Table 11. Data table for use of statins at registration by countries/areas (Figure 2H in the main text) 19](#_Toc118150049)

[Supplementary Table 12. Data table for current smoker at registration by countries/areas (Figure 3A in the main text) 20](#_Toc118150050)

[Supplementary Table 13. Data table for regular alcohol drinker at registration by countries/areas (Figure 3B in the main text) 21](#_Toc118150051)

[Supplementary Table 14. Data table for adherence to balanced diet at registration by countries/areas (Figure 3C in the main text) 22](#_Toc118150052)

[Supplementary Table 15. Data table for physical activity ≥3 times/week at registration by countries/areas (Figure 3D in the main text) 23](#_Toc118150053)

[Supplementary Table 16. Data table for self-monitoring blood glucose ≥once weekly at registration by countries/areas (Figure 3E in the main text) 24](#_Toc118150054)

[Supplementary Table 17. Data table for education by nurses at registration by countries/areas (Figure 3F in the main text) 25](#_Toc118150055)

[Supplementary Table 18. Data table for education by dietitians at registration by countries/areas (Figure 3G in the main text) 26](#_Toc118150056)

[Supplementary Table 19. Data table for education by podiatrists at registration by countries/areas (Figure 3H in the main text) 27](#_Toc118150057)

[Supplementary Table 20. Data table for history of atherosclerotic cardiovascular disease (ASCVD) at registration by countries/areas (Figure 4A in the main text) 28](#_Toc118150058)

[Supplementary Table 21. Data table for history of heart failure hospitalization at registration by countries/areas (Figure 4B in the main text) 29](#_Toc118150059)

[Supplementary Table 22. Data table for estimated glomerular filtration (eGFR) <60 mL/min/1·73m^2^ at registration by countries/areas (Figure 4C in the main text) 30](#_Toc118150060)

[Supplementary Table 23. Data table for any-site cancer at registration by countries/areas (Figure 4D in the main text) 31](#_Toc118150061)

[Supplementary Table 24. Cardiovascular risk profiles and quality of care among patients with type 2 diabetes in Asia, stratified by age group at registration 32](#_Toc118150062)

[Supplementary Table 25. Data table for quality of diabetes care at registration among those with age ≥50 years at registration by countries/areas (Supplementary Figure 1A) 35](#_Toc118150063)

[Supplementary Table 26. Data table for quality of diabetes care at registration among those with a history of ASCVD and/or heart failure at registration by countries/areas (Supplementary Figure 1B) 36](#_Toc118150064)

[Supplementary Table 27. Data table for quality of diabetes care at registration among those with a history of eGFR <60 mL/min/1·73m^2^ at registration by countries/areas (Supplementary Figure 1C) 37](#_Toc118150065)

[Supplementary Table 28. Cardiovascular risk profiles and quality of care among male patients with type 2 diabetes who were either included or excluded (due to missing data) in the analysis 38](#_Toc118150066)

[Supplementary Table 29. Cardiovascular risk profiles and quality of care among female patients with type 2 diabetes who were either included or excluded (due to missing data) in the analysis 40](#_Toc118150067)

[Supplementary Figure 1. Quality of diabetes care at registration, stratified by age and history of comorbidities estimated by proportions with 95% estimates 42](#_Toc118150068)

[References 44](#_Toc118150069)

# **Supplementary Text.** A summary description of the JADE Program^1^

**Rationale and Data Collection**

In patients with type 2 diabetes, the interactions among socio-demographics, genetic factors, lifestyles and cardiometabolic conditions can lead to different trajectories of comorbidities and premature death, depending on access to education, care, and medications. A multicomponent approach is needed to define these risk factors, identify unmet needs, and personalize care in order to achieve better health outcomes.^2,3^

To ease the strain from finite resources, in 1995, the Chinese University of Hong Kong – Prince of Wales Hospital (CUHK-PWH) diabetes team changed the care setting by introducing a weekly nurse-led complication screening session at the ambulatory Diabetes Centre, supervised by diabetologists. Nurses were trained to collect data using a standardized case record form, perform structured assessment (e.g., anthropometric measurements, bedside neuropathy screening, retinopathy screening using a fundus camera), and assist in delivery of protocol-driven care to establish the Hong Kong Diabetes Register (HKDR).^1^ Data were entered into a Microsoft Program for data management and analysis using various algorithms for issuing a personalized report (one to the patient and one to the managing doctor) including treatment targets, attained cardiometabolic risk factors, and cardiorenal complications with recommendations. Patients returned to the Diabetes Centre 4–6 weeks later to collect the personalized reports and attended a 2-hour, nurse-led empowerment class focusing on self-management education and treatment adherence. All patients attending medical clinics in PWH could be referred to the Diabetes Centre for this assessment and education program for quality improvement.^1^ This data-driven integrated care approach was confirmed to improve patient empowerment and patient-provider communication with reduced clinical inertia and treatment non-adherence. In 2000, this care model was adopted by the Hospital Authority which operates all publicly-funded clinics/hospitals in Hong Kong to become a territory-wide program. In a recent analysis of 0.5 billion people with diabetes from 16 high-income countries with nation or territory-wide registers, Hong Kong had the highest decrement of 4% per year for diabetes-related mortality rate in 2000-2016.^4,5^

In 2007, supported by an educational grant, the CUHK-PWH diabetes team designed the *state-of-the-art* web-based JADE portal that incorporates the structured care protocol and HKDR-derived risk algorithms. The web-based JADE Program is an implementation project aimed to share best practices for benchmarking purpose. Upon registration with the JADE portal, the users will have access to the full protocol including the rationale, purpose, definitions, procedures related to the structured assessment, and treatment targets supported by international evidence.^6^ In both the case record form and JADE portal, we have included definitions to help users code responses e.g., types of diabetes, complications, and lifestyle factors. The JADE portal contains reference ranges to prompt users for out-of-range laboratory values. All data are locked at three months after initial data entry and the users will need to request unlocking of the record if data need to be revised or corrected.

As a regional collaborative effort to improve diabetes care in Asia, we launched the JADE Program to assist interested sites out of Hong Kong in establishing a register/database as the foundation of a data-driven integrated diabetes care program.^7,8^ Between 2007 and 2015, investigators in Asia were given a nurse salary to recruit 300-600 patients for structured assessment with entry of anonymized data into the JADE portal. Interested parties were granted password-protected access to the web-based JADE platform which consists of interfaces for users to enter data into a template similar to the case report form used to establish the HKDR. The data were fed to a built-in risk engine including the HKDR risk equations and reporting system. Data were analysed and presented in terms of risk categories based on various combinations of complications and risk factors with 5-year probabilities of clinical events and visualized in the form of bar charts and trend lines. The trends of risk factors control were accompanied by built-in decision support along with a summary of complications, risk factors and recommended/attained targets (HbA_1c_, blood pressure, lipids and body weight) to empower patients and inform clinical decisions (refer to the sample JADE report below). To date, more than 300 sites in 11 Asian countries/regions including mainland China, Hong Kong, India, Indonesia, Malaysia, Philippines, South Korea, Singapore, Taiwan, Thailand, and Vietnam, have registered patients in the JADE Program.

**Definitions of key variables**

During the assessment visit, nurses measured patients’ height, body weight, waist circumference, and vital signs. The case record forms were completed by attending physicians and trained nurses. Blood and urine tests as well as clinical examination of eye and feet were performed as per local practice. All information on medications and comorbidities were validated and checked against patient records and laboratory and imaging tests by attending physicians. The definitions of key variables used in the present analysis are summarized below.

| **Variable** | **Definition** |
| --- | --- |
| Family history of diabetes | Presence of one or more first-degree relative diagnosed with type 2 diabetes |
| HbA_1c_ <7% | Optimal target according to the ADA Standard of Medical Care^9^ |
| BP <130/80 mmHg | Optimal target according to the ADA Standard of Medical Care^10^ |
| LDL-cholesterol <2.6 mmol/L | Risk-based target for high-risk patients according to the ESC/EAS guidelines at time of registration in the JADE Program^11,12^ |
| LDL-cholesterol <1.8 mmol/L | Risk-based target for very high-risk patients according to the ESC/EAS guidelines at the time of registration in the JADE Program^11,12^ |
| Central obesity | Waist circumference ≥90 cm in men and ≥80 cm in women |
| Adherence to balanced diet in last 3 months | 4 coded responses: Yes, No, Occasional, Never |
| Frequency of physical activity (≥30 minutes) in last 3 months | 5 coded responses: No regular activity, <3 times/week, 3-4 times/week, 5 times/week, >5 times/week |
| Self-monitoring in last 3 months | 4 coded responses: < once monthly, at least once monthly, at least once weekly, at least daily |
| ASCVD | - Coronary arterial disease (myocardial infarction, unstable angina, percutaneous coronary intervention, coronary artery bypass graft) - Stroke - Peripheral arterial disease (lower extremity amputation, absent foot pulses with ankle to brachial ratio <0.9 and/or lower limb revascularization) |

**Footnotes:** ASCVD, atherosclerotic cardiovascular disease; BP, blood pressure; LDL, low-density lipoprotein.

**A sample of full JADE report**

# **Supplementary Table 1.** Number of patients by countries/areas and year of registration to the JADE Program

|  | **2007** | **2008** | **2009** | **2010** | **2011** | **2012** | **2013** | **2014** | **2015** |
| --- | --- | --- | --- | --- | --- | --- | --- | --- | --- |
| China | 0 | 0 | 0 | 1,155 | 3,612 | 1,798 | 223 | 3 | 0 |
| Hong Kong | 285 | 2,157 | 2,516 | 5,135 | 3,773 | 2,676 | 2,776 | 2,256 | 2,196 |
| India | 351 | 748 | 1,738 | 2,947 | 2,864 | 3,484 | 9,938 | 13,107 | 5,310 |
| Indonesia | 0 | 0 | 0 | 0 | 0 | 0 | 53 | 2,150 | 288 |
| Korea | 0 | 284 | 585 | 566 | 350 | 22 | 70 | 77 | 257 |
| Malaysia | 0 | 0 | 0 | 0 | 0 | 56 | 1,152 | 124 | 196 |
| Philippines | 72 | 993 | 2,546 | 1,844 | 1,792 | 2,820 | 2,555 | 3,086 | 555 |
| Singapore | 0 | 256 | 20 | 0 | 0 | 164 | 132 | 42 | 0 |
| Taiwan | 0 | 52 | 52 | 8 | 0 | 0 | 1,811 | 919 | 421 |
| Thailand | 0 | 280 | 0 | 0 | 0 | 0 | 433 | 214 | 259 |
| Vietnam | 0 | 0 | 0 | 0 | 0 | 744 | 3,294 | 3,636 | 98 |

# **Supplementary Table 2.** Cardiovascular risk profiles and quality of care among patients with type 2 diabetes from middle-income countries/areas in Asia (China, India, Indonesia, Malaysia, Philippines, Thailand, and Vietnam)

|  | **Women (n=** **36,372)** | | **Men (n=** **40,146)** | |
| --- | --- | --- | --- | --- |
|  | **n** |  | **n** |  |
| **Country/Area, n (%)** | 36,372 |  | 40,146 |  |
| China |  | 2,984 (8·2%) |  | 3,807 (9·5%) |
| India |  | 16,520 (45·4%) |  | 23,967 (59·7%) |
| Indonesia |  | 1,180 (3·2%) |  | 1,311 (3·3%) |
| Malaysia |  | 732 (2·0%) |  | 796 (2·0%) |
| Philippines |  | 9,649 (26·5%) |  | 6,614 (16·5%) |
| Thailand |  | 796 (2·2%) |  | 390 (1·0%) |
| Vietnam |  | 4,511 (12·4%) |  | 3,261 (8·1%) |
| College education, n (%) | 31,415 | 11,434 (36·4%) | 35,318 | 21,377 (60·5%) |
| Family history of diabetes, n (%) | 32,059 | 18,291 (57·1%) | 35,759 | 20,694 (57·9%) |
| Age, years | 36,298 | 57·2±11·7 | 40,094 | 54·7±11·6 |
| Duration of diabetes^¥^, years | 34,044 | 6·0 (2·0, 11·0) | 37,676 | 5·0 (2·0, 11·0) |
| Body mass index, kg/m^2^ | 31,931 | 26·4±5·0 | 36,405 | 26·2±4·3 |
| Waist circumference, cm | 24,482 | 89·1±12·7 | 29,678 | 92·3±11·6 |
| HbA_1c_, % | 27,443 | 8·2±2·0 | 32,010 | 8·3±2·0 |
| Systolic blood pressure, mmHg | 34,231 | 129·4±16·6 | 38,215 | 130·2±16·1 |
| Diastolic blood pressure, mmHg | 34,153 | 78·8±9·0 | 38,123 | 80·3±8·8 |
| Total cholesterol, mmol/L | 25,561 | 4·7 (4·0, 5·5) | 29,249 | 4·5 (3·9, 5·3) |
| Triglyceride^¥^, mmol/L | 26,613 | 1·7 (1·2, 2·2) | 31,320 | 1·7 (1·2, 2·3) |
| HDL-cholesterol, mmol/L | 26,027 | 1·1 (1·0, 1·3) | 30,398 | 1·0 (0·9, 1·2) |
| LDL-cholesterol, mmol/L | 25,943 | 2·7 (2·1, 3·4) | 30,368 | 2·6 (2·0, 3·2) |
| Non-HDL cholesterol, mmol/L | 24,288 | 3·5 (2·8, 4·3) | 27,647 | 3·4 (2·8, 4·2) |
| eGFR, mL/min/1·73m^2^ | 24,636 | 76·5±24·5 | 29,343 | 83·0±24·2 |
| **Cardiometabolic risk factors, n (%)** | | | | |
| Very high CVD risk^γ^ | 34,369 | 34,246 (99·6%) | 38,481 | 38,322 (99·6%) |
| HbA_1c_ <7% | 27,443 | 8,318 (30·3%) | 32,010 | 9,031 (28·2%) |
| Blood pressure <130/80 mmHg | 34,171 | 9,004 (26·3%) | 38,139 | 8,127 (21·3%) |
| Attained risk-based LDL-cholesterol target* | 25,758 | 4,044 (15·7%) | 30,175 | 5,474 (18·1%) |
| General obesity (BMI ≥25 kg/m^2^) | 31,931 | 17,771 (55·7%) | 36,405 | 21,283 (58·5%) |
| Central obesity (waist circumference ≥90 cm in men or ≥80 cm in women) | 24,482 | 19,388 (79·2%) | 29,678 | 17,177 (57·9%) |
| ≥3 treatment targets attained^#^ | 18,667 | 952 (5·1%) | 22,801 | 1,614 (7·1%) |
| Current smoker | 34,286 | 481 (1·4%) | 38,389 | 8,051 (21·0%) |
| Regular alcohol drinker | 34,232 | 97 (0·3%) | 38,269 | 3,239 (8·5%) |
| **Self-reported health habits in last 3 months, n (%)** | | | | |
| Adherence to balanced diet | 33,397 | 27,235 (81·5%) | 36,764 | 29,478 (80·2%) |
| Physical activity ≥3 times/week | 34,057 | 13,354 (39·2%) | 37,670 | 18,485 (49·1%) |
| SMBG ≥ once/week | 29,876 | 7,783 (26·1%) | 32,464 | 8,864 (27·3%) |
| **Exposure to allied health professionals, n (%)** | | | | |
| Education by nurses | 33,404 | 15,653 (46·9%) | 36,569 | 19,004 (52·0%) |
| Education by dietitians | 34,070 | 18,722 (55·0%) | 37,629 | 23,828 (63·3%) |
| Education by podiatrists | 31,759 | 9,873 (31·1%) | 34,195 | 13,036 (38·1%) |
| **Comorbidities, n (%)** | | | | |
| ASCVD | 36,372 | 4,226 (11·6%) | 40,146 | 6,015 (15·0%) |
| Heart failure | 36,372 | 299 (0·8%) | 40,146 | 842 (2·1%) |
| eGFR <60 mL/min/1·73m^2^ | 24,636 | 5,961 (24·2%) | 29,343 | 4,896 (16·7%) |
| Any-site cancer | 36,372 | 374 (1·0%) | 40,146 | 196 (0·5%) |
| **Medication use, n (%)** | | | | |
| Oral glucose-lowering drugs | 36,372 | 31,114 (85·5%) | 40,146 | 34,384 (85·6%) |
| Injectable GLP1-RA | 36,372 | 82 (0·2%) | 40,146 | 110 (0·3%) |
| Insulin | 36,372 | 9,802 (26·9%) | 40,146 | 10,845 (27·0%) |
| Blood pressure-lowering drugs | 36,372 | 20,215 (55·6%) | 40,146 | 20,834 (51·9%) |
| Renin-angiotensin system inhibitors | 29,328 | 10,018 (34·2%) | 33,099 | 10,885 (32·9%) |
| Statin | 29,820 | 11,004 (36·9%) | 33,084 | 12,547 (37·9%) |
| Aspirin | 36,372 | 3,892 (10·7%) | 40,146 | 5,362 (13·4%) |

Footnotes: Data are presented as mean ± standard deviation, ^¥^median (interquartile range) or number (percentage).

^γ^Definition of CV risk was based on the 2016 European Society of Cardiology/European Atherosclerosis Society (ESC/EAS) recommendations in line with the data collection period. ^#^We defined ≥3 treatment targets attained as 1) HbA_1c_<7%, 2) blood pressure <130/80 mmHg, 3) *risk-based LDL-cholesterol target (<2.6 mmol/L if high risk or <1.8 mmol/L if very high-risk), and 4) lack of central obesity (waist circumference <90 cm in men or <80 cm in women).

ASCVD, atherosclerotic cardiovascular disease; BMI, body mass index; eGFR, estimated glomerular filtration rate; GLP1-RA, glucagon-like peptide 1 receptor analogues; HDL, high-density lipoprotein; LDL, low-density lipoprotein; SMBG, self-monitoring blood glucose.

# **Supplementary Table 3.** Cardiovascular risk profiles and quality of care among patients with type 2 diabetes from high-income countries/areas in Asia (Hong Kong SAR, Singapore, South Korea, and Taiwan)

|  | **Women (n=13,452)** | | **Men (n=16,406)** | |
| --- | --- | --- | --- | --- |
|  | **n** |  | **n** |  |
| **Country/Area, n (%)** | 13,452 |  | 16,406 |  |
| Hong Kong |  | 10,648 (79·2%) |  | 13,122 (80·0%) |
| Korea |  | 1,007 (7·5%) |  | 1,204 (7·3%) |
| Singapore |  | 319 (2·4%) |  | 295 (1·8%) |
| Taiwan |  | 1,478 (11·0%) |  | 1,785 (10·9%) |
| College education, n (%) | 13,070 | 1,404 (10·7%) | 15,942 | 22·8% (3639) |
| Family history of diabetes, n (%) | 12,387 | 7,889 (63·7%) | 15,138 | 60·6% (9173) |
| Age, years | 13,452 | 60·6±12·2 | 16,406 | 59·0±11·9 |
| Duration of diabetes^¥^, years | 13,192 | 8·0 (3·0, 15·0) | 16,180 | 7·0 (2·0, 13·0) |
| Body mass index, kg/m^2^ | 13,367 | 25·9±4·6 | 16,302 | 26·0±4·1 |
| Waist circumference, cm | 13,194 | 87·0±11·2 | 16,101 | 91·7±10·6 |
| HbA_1c_, % | 13,363 | 7·5±1·5 | 16,275 | 7·5±1·6 |
| Systolic blood pressure, mmHg | 13,400 | 135·0±19·7 | 16,338 | 133·6±17·9 |
| Diastolic blood pressure, mmHg | 13,388 | 75·9±10·5 | 16,318 | 78·9±10·5 |
| Total cholesterol, mmol/L | 13,015 | 4·6 (4·0, 5·2) | 15,496 | 4·4 (3·8, 5·0) |
| Triglyceride^¥^, mmol/L | 13,257 | 1·3 (0·9, 1·9) | 16,187 | 1·3 (0·9, 1·9) |
| HDL-cholesterol, mmol/L | 13,157 | 1·3 (1·1, 1·6) | 16,065 | 1·2 (1·0, 1·4) |
| LDL-cholesterol, mmol/L | 13,007 | 2·5 (2·0, 3·1) | 15,794 | 2·4 (1·9, 3·0) |
| Non-HDL cholesterol, mmol/L | 12,894 | 3·2 (2·6, 3·8) | 15,372 | 3·1 (2·6, 3·8) |
| eGFR, mL/min/1·73m^2^ | 13,147 | 82·1±25·0 | 16,092 | 80·1±23·5 |
| **Cardiometabolic risk factors, n (%)** | | | | |
| Very high CVD risk^γ^ | 13,368 | 12,986 (97·1%) | 16,314 | 15,966 (97·9%) |
| HbA_1c_ <7% | 13,363 | 5,622 (42·1%) | 16,275 | 7,267 (44·7%) |
| Blood pressure <130/80 mmHg | 13,388 | 5,000 (37·3%) | 16,319 | 5,878 (36·0%) |
| Attained risk-based LDL-cholesterol target* | 12,972 | 2,248 (17·3%) | 15,759 | 3,153 (20·0%) |
| General obesity (BMI ≥25 kg/m^2^) | 13,367 | 7,036 (52·6%) | 16,302 | 9,164 (56·2%) |
| Central obesity (waist circumference ≥90 cm in men or ≥80 cm in women) | 13,194 | 9,796 (74·2%) | 16,101 | 9,074 (56·4%) |
| ≥3 treatment targets attained^#^ | 12,721 | 1,442 (11·3%) | 15,476 | 2,432 (15·7%) |
| Current smoker | 13,417 | 319 (2·4%) | 16,345 | 3,392 (20·8%) |
| Regular alcohol drinker | 13,413 | 68 (0·5%) | 16,327 | 1,262 (7·7%) |
| **Self reported health habits in last 3 months, n (%)** | | | | |
| Adherence to balanced diet | 13,193 | 11,972 (90·7%) | 15,928 | 13,818 (86·8%) |
| Physical activity ≥3 times/week | 13,144 | 6,073 (46·2%) | 15,878 | 7,318 (46·1%) |
| SMBG ≥ once/week | 12,454 | 6,597 (53·0%) | 15,180 | 8,119 (53·5%) |
| **Exposure to allied health professionals, n (%)** | | | | |
| Education by nurses | 13,347 | 8,546 (64·0%) | 16,282 | 10,002 (61·4%) |
| Education by dietitians | 13,347 | 9,253 (69·3%) | 16,286 | 10,541 (64·7%) |
| Education by podiatrists | 13,101 | 3,195 (24·4%) | 16,013 | 3,487 (21·8%) |
| **Comorbidities, n (%)** | | | | |
| ASCVD | 13,452 | 2,163 (16·1%) | 16,406 | 3,606 (22·0%) |
| Heart failure | 13,452 | 342 (2·5%) | 16,406 | 441 (2·7%) |
| eGFR <60 mL/min/1·73m^2^ | 13,147 | 2,482 (18·9%) | 16,092 | 3,110 (19·3%) |
| Any-site cancer | 13,452 | 851 (6·3%) | 16,406 | 725 (4·4%) |
| **Medication use, n (%)** | | | | |
| Oral glucose-lowering drugs | 13,452 | 11,530 (85·7%) | 16,406 | 14,037 (85·6%) |
| Injectable GLP1-RA | 13,452 | 23 (0·2%) | 16,406 | 35 (0·2%) |
| Insulin | 13,452 | 3,657 (27·2%) | 16,406 | 4,142 (25·2%) |
| Blood pressure-lowering drugs | 13,452 | 9,036 (67·2%) | 16,406 | 10,944 (66·7%) |
| Renin-angiotensin system inhibitors | 12,795 | 5,936 (46·4%) | 15,562 | 7,587 (48·8%) |
| Statin | 12,238 | 5,959 (48·7%) | 14,842 | 6,971 (47·0%) |
| Aspirin | 1,3452 | 2,458 (18·3%) | 16406 | 3,953 (24·1%) |

Footnotes: Data are presented as mean ± standard deviation, ^¥^median (interquartile range) or number (percentage).

^γ^Definition of CV risk was based on the 2016 European Society of Cardiology/European Atherosclerosis Society (ESC/EAS) recommendations in line with the data collection period. ^#^We defined ≥3 treatment targets attained as 1) HbA_1c_<7%, 2) blood pressure <130/80 mmHg, 3) *risk-based LDL-cholesterol target (<2.6 mmol/L if high risk or <1.8 mmol/L if very high-risk), and 4) lack of central obesity (waist circumference <90 cm in men or <80 cm in women).

ASCVD, atherosclerotic cardiovascular disease; BMI, body mass index; eGFR, estimated glomerular filtration rate; GLP1-RA, glucagon-like peptide 1 receptor analogues; HDL, high-density lipoprotein; LDL, low-density lipoprotein; SMBG, self-monitoring blood glucose.

# **Supplementary Table 4.** Data table for HbA_1c_ <7% at registration by countries/areas (Figure 2A in the main text)

| **Sex** | **Country/region** | **Yes** | **No** | **Total** | **Proportion** | **95% CI, lower** | **95% CI, upper** |
| --- | --- | --- | --- | --- | --- | --- | --- |
| F | China | 1102 | 1728 | 2830 | 38·9 | 37·1 | 40·8 |
| F | Hong Kong | 4415 | 6188 | 10603 | 41·6 | 40·7 | 42·6 |
| F | India | 2815 | 10440 | 13255 | 21·2 | 20·5 | 21·9 |
| F | Indonesia | 193 | 520 | 713 | 27·1 | 23·8 | 30·5 |
| F | Korea | 525 | 466 | 991 | 53·0 | 49·8 | 56·1 |
| F | Malaysia | 258 | 433 | 691 | 37·3 | 33·7 | 41·1 |
| F | Philippines | 2102 | 2977 | 5079 | 41·4 | 40·0 | 42·8 |
| F | Singapore | 93 | 216 | 309 | 30·1 | 25·0 | 35·5 |
| F | Taiwan | 589 | 871 | 1460 | 40·3 | 37·8 | 42·9 |
| F | Thailand | 228 | 557 | 785 | 29·0 | 25·9 | 32·4 |
| F | Vietnam | 1620 | 2470 | 4090 | 39·6 | 38·1 | 41·1 |
| M | China | 1315 | 2270 | 3585 | 36·7 | 35·1 | 38·3 |
| M | Hong Kong | 5725 | 7322 | 13047 | 43·9 | 43·0 | 44·7 |
| M | India | 4045 | 15635 | 19680 | 20·6 | 20·0 | 21·1 |
| M | Indonesia | 281 | 579 | 860 | 32·7 | 29·5 | 35·9 |
| M | Korea | 686 | 501 | 1187 | 57·8 | 54·9 | 60·6 |
| M | Malaysia | 290 | 481 | 771 | 37·6 | 34·2 | 41·1 |
| M | Philippines | 1606 | 2156 | 3762 | 42·7 | 41·1 | 44·3 |
| M | Singapore | 77 | 206 | 283 | 27·2 | 22·1 | 32·8 |
| M | Taiwan | 779 | 979 | 1758 | 44·3 | 42·0 | 46·7 |
| M | Thailand | 144 | 239 | 383 | 37·6 | 32·7 | 42·7 |
| M | Vietnam | 1350 | 1619 | 2969 | 45·5 | 43·7 | 47·3 |

# **Supplementary Table 5.** Data table for blood pressure <130/80 mmHg at registration by countries/areas (Figure 2B in the main text)

| **Sex** | **Country/region** | **Yes** | **No** | **Total** | **Proportion** | **95% CI, lower** | **95% CI, upper** |
| --- | --- | --- | --- | --- | --- | --- | --- |
| F | China | 1100 | 1820 | 2920 | 37·7 | 35·9 | 39·5 |
| F | Hong Kong | 3930 | 6696 | 10626 | 37·0 | 36·1 | 37·9 |
| F | India | 3499 | 12678 | 16177 | 21·6 | 21·0 | 22·3 |
| F | Indonesia | 190 | 694 | 884 | 21·5 | 18·8 | 24·4 |
| F | Korea | 563 | 421 | 984 | 57·2 | 54·1 | 60·3 |
| F | Malaysia | 158 | 550 | 708 | 22·3 | 19·3 | 25·6 |
| F | Philippines | 2175 | 6189 | 8364 | 26·0 | 25·1 | 27·0 |
| F | Singapore | 85 | 218 | 303 | 28·1 | 23·1 | 33·5 |
| F | Taiwan | 422 | 1053 | 1475 | 28·6 | 26·3 | 31·0 |
| F | Thailand | 231 | 562 | 793 | 29·1 | 26·0 | 32·4 |
| F | Vietnam | 1651 | 2674 | 4325 | 38·2 | 36·7 | 39·6 |
| M | China | 1141 | 2555 | 3696 | 30·9 | 29·4 | 32·4 |
| M | Hong Kong | 4683 | 8398 | 13081 | 35·8 | 35·0 | 36·6 |
| M | India | 3962 | 19414 | 23376 | 16·9 | 16·5 | 17·4 |
| M | Indonesia | 244 | 784 | 1028 | 23·7 | 21·2 | 26·5 |
| M | Korea | 630 | 548 | 1178 | 53·5 | 50·6 | 56·4 |
| M | Malaysia | 169 | 608 | 777 | 21·8 | 18·9 | 24·8 |
| M | Philippines | 1336 | 4418 | 5754 | 23·2 | 22·1 | 24·3 |
| M | Singapore | 59 | 221 | 280 | 21·1 | 16·4 | 26·3 |
| M | Taiwan | 506 | 1274 | 1780 | 28·4 | 26·3 | 30·6 |
| M | Thailand | 112 | 277 | 389 | 28·8 | 24·3 | 33·6 |
| M | Vietnam | 1163 | 1956 | 3119 | 37·3 | 35·6 | 39·0 |

# **Supplementary Table 6.** Data table for risk-based LDL-cholesterol target at registration by countries/areas (Figure 2C in the main text)

| **Sex** | **Country/region** | **Yes** | **No** | **Total** | **Proportion** | **95% CI, lower** | **95% CI, upper** |
| --- | --- | --- | --- | --- | --- | --- | --- |
| F | China | 279 | 2461 | 2740 | 10·2 | 9·1 | 11·4 |
| F | Hong Kong | 1799 | 8631 | 10430 | 17·2 | 16·5 | 18·0 |
| F | India | 2190 | 10286 | 12476 | 17·6 | 16·9 | 18·2 |
| F | Indonesia | 80 | 639 | 719 | 11·1 | 8·9 | 13·7 |
| F | Korea | 181 | 667 | 848 | 21·3 | 18·6 | 24·3 |
| F | Malaysia | 129 | 550 | 679 | 19·0 | 16·1 | 22·2 |
| F | Philippines | 579 | 3748 | 4327 | 13·4 | 12·4 | 14·4 |
| F | Singapore | 31 | 264 | 295 | 10·5 | 7·3 | 14·6 |
| F | Taiwan | 237 | 1162 | 1399 | 16·9 | 15·0 | 19·0 |
| F | Thailand | 121 | 660 | 781 | 15·5 | 13·0 | 18·2 |
| F | Vietnam | 666 | 3370 | 4036 | 16·5 | 15·4 | 17·7 |
| M | China | 378 | 3156 | 3534 | 10·7 | 9·7 | 11·8 |
| M | Hong Kong | 2541 | 10204 | 12745 | 19·9 | 19·2 | 20·6 |
| M | India | 3729 | 15062 | 18791 | 19·8 | 19·3 | 20·4 |
| M | Indonesia | 106 | 779 | 885 | 12·0 | 9·9 | 14·3 |
| M | Korea | 269 | 781 | 1050 | 25·6 | 23·0 | 28·4 |
| M | Malaysia | 168 | 581 | 749 | 22·4 | 19·5 | 25·6 |
| M | Philippines | 431 | 2484 | 2915 | 14·8 | 13·5 | 16·1 |
| M | Singapore | 43 | 228 | 271 | 15·9 | 11·7 | 20·8 |
| M | Taiwan | 300 | 1393 | 1693 | 17·7 | 15·9 | 19·6 |
| M | Thailand | 82 | 297 | 379 | 21·6 | 17·6 | 26·1 |
| M | Vietnam | 580 | 2342 | 2922 | 19·8 | 18·4 | 21·3 |

# **Supplementary Table 7.** Data table for waist circumference below target at registration by countries/areas (Figure 2D in the main text)

| **Sex** | **Country/region** | **Yes** | **No** | **Total** | **Proportion** | **95% CI, lower** | **95% CI, upper** |
| --- | --- | --- | --- | --- | --- | --- | --- |
| F | China | 499 | 1958 | 2457 | 20·3 | 18·7 | 22·0 |
| F | Hong Kong | 2851 | 7750 | 10601 | 26·9 | 26·1 | 27·7 |
| F | India | 2925 | 10772 | 13697 | 21·4 | 20·7 | 22·1 |
| F | Indonesia | 133 | 641 | 774 | 17·2 | 14·6 | 20·0 |
| F | Korea | 252 | 712 | 964 | 26·1 | 23·4 | 29·0 |
| F | Malaysia | 92 | 622 | 714 | 12·9 | 10·5 | 15·6 |
| F | Philippines | 363 | 1483 | 1846 | 19·7 | 17·9 | 21·6 |
| F | Singapore | 23 | 147 | 170 | 13·5 | 8·8 | 19·6 |
| F | Taiwan | 272 | 1187 | 1459 | 18·6 | 16·7 | 20·7 |
| F | Thailand | 148 | 637 | 785 | 18·9 | 16·2 | 21·8 |
| F | Vietnam | 934 | 3275 | 4209 | 22·2 | 20·9 | 23·5 |
| M | China | 1303 | 1827 | 3130 | 41·6 | 39·9 | 43·4 |
| M | Hong Kong | 5688 | 7343 | 13031 | 43·6 | 42·8 | 44·5 |
| M | India | 8312 | 11662 | 19974 | 41·6 | 40·9 | 42·3 |
| M | Indonesia | 296 | 588 | 884 | 33·5 | 30·4 | 36·7 |
| M | Korea | 637 | 486 | 1123 | 56·7 | 53·8 | 59·6 |
| M | Malaysia | 203 | 574 | 777 | 26·1 | 23·1 | 29·4 |
| M | Philippines | 721 | 818 | 1539 | 46·8 | 44·3 | 49·4 |
| M | Singapore | 47 | 134 | 181 | 26·0 | 19·7 | 33·0 |
| M | Taiwan | 655 | 1111 | 1766 | 37·1 | 34·8 | 39·4 |
| M | Thailand | 144 | 242 | 386 | 37·3 | 32·5 | 42·3 |
| M | Vietnam | 1522 | 1466 | 2988 | 50·9 | 49·1 | 52·7 |

# **Supplementary Table 8.** Data table for ≥3 treatment targets at registration by countries/areas (Figure 2E in the main text)

| **Sex** | **Country/region** | **Yes** | **No** | **Total** | **Proportion** | **95% CI, lower** | **95% CI, upper** |
| --- | --- | --- | --- | --- | --- | --- | --- |
| F | China | 170 | 2063 | 2233 | 7·6 | 6·5 | 8·8 |
| F | Hong Kong | 1181 | 9204 | 10385 | 11·4 | 10·8 | 12·0 |
| F | India | 283 | 9638 | 9921 | 2·9 | 2·5 | 3·2 |
| F | Indonesia | 22 | 498 | 520 | 4·2 | 2·7 | 6·3 |
| F | Korea | 151 | 642 | 793 | 19·0 | 16·4 | 22·0 |
| F | Malaysia | 37 | 602 | 639 | 5·8 | 4·1 | 7·9 |
| F | Philippines | 72 | 925 | 997 | 7·2 | 5·7 | 9·0 |
| F | Singapore | 6 | 152 | 158 | 3·8 | 1·4 | 8·1 |
| F | Taiwan | 104 | 1281 | 1385 | 7·5 | 6·2 | 9·0 |
| F | Thailand | 52 | 716 | 768 | 6·8 | 5·1 | 8·8 |
| F | Vietnam | 316 | 3273 | 3589 | 8·8 | 7·9 | 9·8 |
| M | China | 238 | 2612 | 2850 | 8·4 | 7·4 | 9·4 |
| M | Hong Kong | 1955 | 10726 | 12681 | 15·4 | 14·8 | 16·1 |
| M | India | 744 | 14146 | 14890 | 5·0 | 4·7 | 5·4 |
| M | Indonesia | 44 | 595 | 639 | 6·9 | 5·0 | 9·1 |
| M | Korea | 279 | 667 | 946 | 29·5 | 26·6 | 32·5 |
| M | Malaysia | 59 | 657 | 716 | 8·2 | 6·3 | 10·5 |
| M | Philippines | 75 | 696 | 771 | 9·7 | 7·7 | 12·0 |
| M | Singapore | 11 | 163 | 174 | 6·3 | 3·2 | 11·0 |
| M | Taiwan | 187 | 1488 | 1675 | 11·2 | 9·7 | 12·8 |
| M | Thailand | 36 | 336 | 372 | 9·7 | 6·9 | 13·1 |
| M | Vietnam | 418 | 2145 | 2563 | 16·3 | 14·9 | 17·8 |

# **Supplementary Table 9.** Data table for BMI <25 kg/m^2^ at registration by countries/areas (Figure 2F in the main text)

| **Sex** | **Country/region** | **Yes** | **No** | **Total** | **Proportion** | **95% CI, lower** | **95% CI, upper** |
| --- | --- | --- | --- | --- | --- | --- | --- |
| F | China | 1686 | 1259 | 2945 | 57·2 | 55·4 | 59·0 |
| F | Hong Kong | 5054 | 5540 | 10594 | 47·7 | 46·8 | 48·7 |
| F | India | 5298 | 10565 | 15863 | 33·4 | 32·7 | 34·1 |
| F | Indonesia | 413 | 429 | 842 | 49·0 | 45·6 | 52·5 |
| F | Korea | 544 | 455 | 999 | 54·5 | 51·3 | 57·6 |
| F | Malaysia | 185 | 518 | 703 | 26·3 | 23·1 | 29·7 |
| F | Philippines | 3393 | 3048 | 6441 | 52·7 | 51·5 | 53·9 |
| F | Singapore | 116 | 187 | 303 | 38·3 | 32·8 | 44·0 |
| F | Taiwan | 617 | 854 | 1471 | 41·9 | 39·4 | 44·5 |
| F | Thailand | 359 | 426 | 785 | 45·7 | 42·2 | 49·3 |
| F | Vietnam | 2826 | 1526 | 4352 | 64·9 | 63·5 | 66·4 |
| M | China | 1871 | 1877 | 3748 | 49·9 | 48·3 | 51·5 |
| M | Hong Kong | 5616 | 7428 | 13044 | 43·1 | 42·2 | 43·9 |
| M | India | 8343 | 14616 | 22959 | 36·3 | 35·7 | 37·0 |
| M | Indonesia | 434 | 561 | 995 | 43·6 | 40·5 | 46·8 |
| M | Korea | 698 | 500 | 1198 | 58·3 | 55·4 | 61·1 |
| M | Malaysia | 248 | 529 | 777 | 31·9 | 28·6 | 35·3 |
| M | Philippines | 2084 | 2320 | 4404 | 47·3 | 45·8 | 48·8 |
| M | Singapore | 92 | 184 | 276 | 33·3 | 27·8 | 39·2 |
| M | Taiwan | 732 | 1052 | 1784 | 41·0 | 38·7 | 43·4 |
| M | Thailand | 154 | 233 | 387 | 39·8 | 34·9 | 44·9 |
| M | Vietnam | 1988 | 1147 | 3135 | 63·4 | 61·7 | 65·1 |

# **Supplementary Table 10.** Data table for use of RAS inhibitors at registration by countries/areas (Figure 2G in the main text)

| **Sex** | **Country/region** | **Yes** | **No** | **Total** | **Proportion** | **95% CI, lower** | **95% CI, upper** |
| --- | --- | --- | --- | --- | --- | --- | --- |
| F | China | 605 | 1745 | 2350 | 25·7 | 24·0 | 27·6 |
| F | Hong Kong | 4640 | 5674 | 10314 | 45·0 | 44·0 | 46·0 |
| F | India | 4616 | 8819 | 13435 | 34·4 | 33·6 | 35·2 |
| F | Indonesia | 229 | 793 | 1022 | 22·4 | 19·9 | 25·1 |
| F | Korea | 441 | 450 | 891 | 49·5 | 46·2 | 52·8 |
| F | Malaysia | 395 | 303 | 698 | 56·6 | 52·8 | 60·3 |
| F | Philippines | 1833 | 5445 | 7278 | 25·2 | 24·2 | 26·2 |
| F | Singapore | 101 | 115 | 216 | 46·8 | 40·0 | 53·6 |
| F | Taiwan | 754 | 620 | 1374 | 54·9 | 52·2 | 57·5 |
| F | Thailand | 432 | 320 | 752 | 57·4 | 53·8 | 61·0 |
| F | Vietnam | 1908 | 1885 | 3793 | 50·3 | 48·7 | 51·9 |
| M | China | 694 | 2445 | 3139 | 22·1 | 20·7 | 23·6 |
| M | Hong Kong | 6090 | 6536 | 12626 | 48·2 | 47·4 | 49·1 |
| M | India | 6786 | 12921 | 19707 | 34·4 | 33·8 | 35·1 |
| M | Indonesia | 329 | 851 | 1180 | 27·9 | 25·3 | 30·5 |
| M | Korea | 524 | 547 | 1071 | 48·9 | 45·9 | 52·0 |
| M | Malaysia | 507 | 243 | 750 | 67·6 | 64·1 | 70·9 |
| M | Philippines | 1110 | 4019 | 5129 | 21·6 | 20·5 | 22·8 |
| M | Singapore | 114 | 96 | 210 | 54·3 | 47·3 | 61·2 |
| M | Taiwan | 859 | 796 | 1655 | 51·9 | 49·5 | 54·3 |
| M | Thailand | 221 | 145 | 366 | 60·4 | 55·2 | 65·4 |
| M | Vietnam | 1238 | 1590 | 2828 | 43·8 | 41·9 | 45·6 |

# **Supplementary Table 11.** Data table for use of statins at registration by countries/areas (Figure 2H in the main text)

| **Sex** | **Country/region** | **Yes** | **No** | **Total** | **Proportion** | **95% CI, lower** | **95% CI, upper** |
| --- | --- | --- | --- | --- | --- | --- | --- |
| F | China | 767 | 1708 | 2475 | 31·0 | 29·2 | 32·9 |
| F | Hong Kong | 4668 | 5284 | 9952 | 46·9 | 45·9 | 47·9 |
| F | India | 5233 | 8600 | 13833 | 37·8 | 37·0 | 38·6 |
| F | Indonesia | 255 | 748 | 1003 | 25·4 | 22·8 | 28·2 |
| F | Korea | 510 | 341 | 851 | 59·9 | 56·5 | 63·2 |
| F | Malaysia | 515 | 120 | 635 | 81·1 | 77·8 | 84·1 |
| F | Philippines | 1834 | 5761 | 7595 | 24·1 | 23·2 | 25·1 |
| F | Singapore | 106 | 116 | 222 | 47·7 | 41·0 | 54·5 |
| F | Taiwan | 675 | 538 | 1213 | 55·6 | 52·8 | 58·5 |
| F | Thailand | 551 | 190 | 741 | 74·4 | 71·1 | 77·5 |
| F | Vietnam | 1849 | 1689 | 3538 | 52·3 | 50·6 | 53·9 |
| M | China | 919 | 2306 | 3225 | 28·5 | 26·9 | 30·1 |
| M | Hong Kong | 5646 | 6521 | 12167 | 46·4 | 45·5 | 47·3 |
| M | India | 8251 | 11575 | 19826 | 41·6 | 40·9 | 42·3 |
| M | Indonesia | 319 | 767 | 1086 | 29·4 | 26·7 | 32·2 |
| M | Korea | 544 | 492 | 1036 | 52·5 | 49·4 | 55·6 |
| M | Malaysia | 584 | 114 | 698 | 83·7 | 80·7 | 86·3 |
| M | Philippines | 1003 | 4313 | 5316 | 18·9 | 17·8 | 19·9 |
| M | Singapore | 125 | 97 | 222 | 56·3 | 49·5 | 62·9 |
| M | Taiwan | 656 | 761 | 1417 | 46·3 | 43·7 | 48·9 |
| M | Thailand | 280 | 84 | 364 | 76·9 | 72·2 | 81·2 |
| M | Vietnam | 1191 | 1378 | 2569 | 46·4 | 44·4 | 48·3 |

# **Supplementary Table 12.** Data table for current smoker at registration by countries/areas (Figure 3A in the main text)

| **Sex** | **Country/region** | **Yes** | **No** | **Total** | **Proportion** | **95% CI, lower** | **95% CI, upper** |
| --- | --- | --- | --- | --- | --- | --- | --- |
| F | China | 55 | 2881 | 2936 | 1·9 | 1·4 | 2·4 |
| F | Hong Kong | 257 | 10371 | 10628 | 2·4 | 2·1 | 2·7 |
| F | India | 89 | 15635 | 15724 | 0·6 | 0·5 | 0·7 |
| F | Indonesia | 19 | 931 | 950 | 2·0 | 1·2 | 3·1 |
| F | Korea | 29 | 973 | 1002 | 2·9 | 1·9 | 4·1 |
| F | Malaysia | 4 | 718 | 722 | 0·6 | 0·2 | 1·4 |
| F | Philippines | 270 | 8484 | 8754 | 3·1 | 2·7 | 3·5 |
| F | Singapore | 0 | 309 | 309 | 0·0 | 0·0 | 1·2 |
| F | Taiwan | 33 | 1445 | 1478 | 2·2 | 1·5 | 3·1 |
| F | Thailand | 6 | 790 | 796 | 0·8 | 0·3 | 1·6 |
| F | Vietnam | 38 | 4366 | 4404 | 0·9 | 0·6 | 1·2 |
| M | China | 1469 | 2278 | 3747 | 39·2 | 37·6 | 40·8 |
| M | Hong Kong | 2566 | 10511 | 13077 | 19·6 | 18·9 | 20·3 |
| M | India | 4288 | 19005 | 23293 | 18·4 | 17·9 | 18·9 |
| M | Indonesia | 259 | 826 | 1085 | 23·9 | 21·4 | 26·5 |
| M | Korea | 372 | 829 | 1201 | 31·0 | 28·4 | 33·7 |
| M | Malaysia | 120 | 674 | 794 | 15·1 | 12·7 | 17·8 |
| M | Philippines | 933 | 4959 | 5892 | 15·8 | 14·9 | 16·8 |
| M | Singapore | 47 | 236 | 283 | 16·6 | 12·5 | 21·5 |
| M | Taiwan | 407 | 1377 | 1784 | 22·8 | 20·9 | 24·8 |
| M | Thailand | 47 | 342 | 389 | 12·1 | 9·0 | 15·7 |
| M | Vietnam | 935 | 2254 | 3189 | 29·3 | 27·7 | 30·9 |

# **Supplementary Table 13.** Data table for regular alcohol drinker at registration by countries/areas (Figure 3B in the main text)

| **Sex** | **Country/region** | **Yes** | **No** | **Total** | **Proportion** | **95% CI, lower** | **95% CI, upper** |
| --- | --- | --- | --- | --- | --- | --- | --- |
| F | China | 28 | 2915 | 2943 | 1·0 | 0·6 | 1·4 |
| F | Hong Kong | 47 | 10578 | 10625 | 0·4 | 0·3 | 0·6 |
| F | India | 35 | 15661 | 15696 | 0·2 | 0·2 | 0·3 |
| F | Indonesia | 0 | 941 | 941 | 0·0 | 0·0 | 0·4 |
| F | Korea | 17 | 985 | 1002 | 1·7 | 1·0 | 2·7 |
| F | Malaysia | 0 | 720 | 720 | 0·0 | 0·0 | 0·5 |
| F | Philippines | 21 | 8729 | 8750 | 0·2 | 0·1 | 0·4 |
| F | Singapore | 1 | 307 | 308 | 0·3 | 0·0 | 1·8 |
| F | Taiwan | 3 | 1475 | 1478 | 0·2 | 0·0 | 0·6 |
| F | Thailand | 2 | 792 | 794 | 0·3 | 0·0 | 0·9 |
| F | Vietnam | 11 | 4377 | 4388 | 0·3 | 0·1 | 0·4 |
| M | China | 663 | 3093 | 3756 | 17·7 | 16·4 | 18·9 |
| M | Hong Kong | 857 | 12208 | 13065 | 6·6 | 6·1 | 7·0 |
| M | India | 2128 | 21078 | 23206 | 9·2 | 8·8 | 9·5 |
| M | Indonesia | 2 | 1074 | 1076 | 0·2 | 0·0 | 0·7 |
| M | Korea | 298 | 904 | 1202 | 24·8 | 22·4 | 27·3 |
| M | Malaysia | 25 | 764 | 789 | 3·2 | 2·1 | 4·6 |
| M | Philippines | 190 | 5684 | 5874 | 3·2 | 2·8 | 3·7 |
| M | Singapore | 9 | 270 | 279 | 3·2 | 1·5 | 6·0 |
| M | Taiwan | 98 | 1683 | 1781 | 5·5 | 4·5 | 6·7 |
| M | Thailand | 15 | 371 | 386 | 3·9 | 2·2 | 6·3 |
| M | Vietnam | 216 | 2966 | 3182 | 6·8 | 5·9 | 7·7 |

# **Supplementary Table 14.** Data table for adherence to balanced diet at registration by countries/areas (Figure 3C in the main text)

| **Sex** | **Country/region** | **Yes** | **No** | **Total** | **Proportion** | **95% CI, lower** | **95% CI, upper** |
| --- | --- | --- | --- | --- | --- | --- | --- |
| F | China | 2316 | 618 | 2934 | 78·9 | 77·4 | 80·4 |
| F | Hong Kong | 9534 | 880 | 10414 | 91·5 | 91·0 | 92·1 |
| F | India | 12480 | 2727 | 15207 | 82·1 | 81·4 | 82·7 |
| F | Indonesia | 666 | 251 | 917 | 72·6 | 69·6 | 75·5 |
| F | Korea | 883 | 115 | 998 | 88·5 | 86·3 | 90·4 |
| F | Malaysia | 551 | 159 | 710 | 77·6 | 74·4 | 80·6 |
| F | Philippines | 6674 | 1811 | 8485 | 78·7 | 77·8 | 79·5 |
| F | Singapore | 281 | 27 | 308 | 91·2 | 87·5 | 94·1 |
| F | Taiwan | 1274 | 199 | 1473 | 86·5 | 84·6 | 88·2 |
| F | Thailand | 696 | 97 | 793 | 87·8 | 85·3 | 90·0 |
| F | Vietnam | 3852 | 499 | 4351 | 88·5 | 87·5 | 89·5 |
| M | China | 2722 | 1006 | 3728 | 73·0 | 71·6 | 74·4 |
| M | Hong Kong | 11070 | 1604 | 12674 | 87·3 | 86·8 | 87·9 |
| M | India | 18102 | 3934 | 22036 | 82·1 | 81·6 | 82·7 |
| M | Indonesia | 722 | 332 | 1054 | 68·5 | 65·6 | 71·3 |
| M | Korea | 1051 | 143 | 1194 | 88·0 | 86·0 | 89·8 |
| M | Malaysia | 573 | 204 | 777 | 73·7 | 70·5 | 76·8 |
| M | Philippines | 4345 | 1297 | 5642 | 77·0 | 75·9 | 78·1 |
| M | Singapore | 253 | 32 | 285 | 88·8 | 84·5 | 92·2 |
| M | Taiwan | 1444 | 331 | 1775 | 81·4 | 79·5 | 83·1 |
| M | Thailand | 342 | 47 | 389 | 87·9 | 84·3 | 91·0 |
| M | Vietnam | 2672 | 466 | 3138 | 85·1 | 83·9 | 86·4 |

# **Supplementary Table 15.** Data table for physical activity ≥3 times/week at registration by countries/areas (Figure 3D in the main text)

| **Sex** | **Country/region** | **Yes** | **No** | **Total** | **Proportion** | **95% CI, lower** | **95% CI, upper** |
| --- | --- | --- | --- | --- | --- | --- | --- |
| F | China | 1219 | 1729 | 2948 | 41·4 | 39·6 | 43·2 |
| F | Hong Kong | 4768 | 5592 | 10360 | 46·0 | 45·1 | 47·0 |
| F | India | 5371 | 10313 | 15684 | 34·2 | 33·5 | 35·0 |
| F | Indonesia | 159 | 762 | 921 | 17·3 | 14·9 | 19·9 |
| F | Korea | 516 | 485 | 1001 | 51·5 | 48·4 | 54·7 |
| F | Malaysia | 208 | 509 | 717 | 29·0 | 25·7 | 32·5 |
| F | Philippines | 3291 | 5324 | 8615 | 38·2 | 37·2 | 39·2 |
| F | Singapore | 87 | 221 | 308 | 28·2 | 23·3 | 33·6 |
| F | Taiwan | 702 | 773 | 1475 | 47·6 | 45·0 | 50·2 |
| F | Thailand | 326 | 469 | 795 | 41·0 | 37·6 | 44·5 |
| F | Vietnam | 2780 | 1597 | 4377 | 63·5 | 62·1 | 64·9 |
| M | China | 1584 | 2156 | 3740 | 42·4 | 40·8 | 44·0 |
| M | Hong Kong | 5610 | 7009 | 12619 | 44·5 | 43·6 | 45·3 |
| M | India | 11341 | 11409 | 22750 | 49·9 | 49·2 | 50·5 |
| M | Indonesia | 260 | 802 | 1062 | 24·5 | 21·9 | 27·2 |
| M | Korea | 731 | 468 | 1199 | 61·0 | 58·1 | 63·7 |
| M | Malaysia | 279 | 507 | 786 | 35·5 | 32·1 | 39·0 |
| M | Philippines | 2586 | 3211 | 5797 | 44·6 | 43·3 | 45·9 |
| M | Singapore | 80 | 197 | 277 | 28·9 | 23·6 | 34·6 |
| M | Taiwan | 897 | 886 | 1783 | 50·3 | 48·0 | 52·7 |
| M | Thailand | 175 | 214 | 389 | 45·0 | 40·0 | 50·1 |
| M | Vietnam | 2260 | 886 | 3146 | 71·8 | 70·2 | 73·4 |

# **Supplementary Table 16.** Data table for self-monitoring blood glucose ≥once weekly at registration by countries/areas (Figure 3E in the main text)

| **Sex** | **Country/region** | **Yes** | **No** | **Total** | **Proportion** | **95% CI, lower** | **95% CI, upper** |
| --- | --- | --- | --- | --- | --- | --- | --- |
| F | China | 875 | 1693 | 2568 | 34·1 | 32·2 | 35·9 |
| F | Hong Kong | 5235 | 4616 | 9851 | 53·1 | 52·2 | 54·1 |
| F | India | 2945 | 10543 | 13488 | 21·8 | 21·1 | 22·5 |
| F | Indonesia | 247 | 608 | 855 | 28·9 | 25·9 | 32·1 |
| F | Korea | 590 | 305 | 895 | 65·9 | 62·7 | 69·0 |
| F | Malaysia | 273 | 378 | 651 | 41·9 | 38·1 | 45·8 |
| F | Philippines | 2467 | 5237 | 7704 | 32·0 | 31·0 | 33·1 |
| F | Singapore | 100 | 195 | 295 | 33·9 | 28·5 | 39·6 |
| F | Taiwan | 672 | 741 | 1413 | 47·6 | 44·9 | 50·2 |
| F | Thailand | 76 | 639 | 715 | 10·6 | 8·5 | 13·1 |
| F | Vietnam | 900 | 2995 | 3895 | 23·1 | 21·8 | 24·5 |
| M | China | 1113 | 2102 | 3215 | 34·6 | 33·0 | 36·3 |
| M | Hong Kong | 6519 | 5609 | 12128 | 53·8 | 52·9 | 54·6 |
| M | India | 5027 | 14539 | 19566 | 25·7 | 25·1 | 26·3 |
| M | Indonesia | 256 | 705 | 961 | 26·6 | 23·9 | 29·6 |
| M | Korea | 743 | 329 | 1072 | 69·3 | 66·5 | 72·1 |
| M | Malaysia | 319 | 400 | 719 | 44·4 | 40·7 | 48·1 |
| M | Philippines | 1343 | 3510 | 4853 | 27·7 | 26·4 | 29·0 |
| M | Singapore | 102 | 169 | 271 | 37·6 | 31·8 | 43·7 |
| M | Taiwan | 755 | 954 | 1709 | 44·2 | 41·8 | 46·6 |
| M | Thailand | 63 | 277 | 340 | 18·5 | 14·5 | 23·1 |
| M | Vietnam | 743 | 2067 | 2810 | 26·4 | 24·8 | 28·1 |

# **Supplementary Table 17.** Data table for education by nurses at registration by countries/areas (Figure 3F in the main text)

| **Sex** | **Country/region** | **Yes** | **No** | **Total** | **Proportion** | **95% CI, lower** | **95% CI, upper** |
| --- | --- | --- | --- | --- | --- | --- | --- |
| F | China | 83 | 2771 | 2854 | 2·9 | 2·3 | 3·6 |
| F | Hong Kong | 6749 | 3817 | 10566 | 63·9 | 63·0 | 64·8 |
| F | India | 10212 | 5063 | 15275 | 66·9 | 66·1 | 67·6 |
| F | Indonesia | 411 | 521 | 932 | 44·1 | 40·9 | 47·4 |
| F | Korea | 706 | 290 | 996 | 70·9 | 68·0 | 73·7 |
| F | Malaysia | 253 | 468 | 721 | 35·1 | 31·6 | 38·7 |
| F | Philippines | 3409 | 5255 | 8664 | 39·3 | 38·3 | 40·4 |
| F | Singapore | 126 | 182 | 308 | 40·9 | 35·4 | 46·6 |
| F | Taiwan | 965 | 512 | 1477 | 65·3 | 62·8 | 67·8 |
| F | Thailand | 687 | 107 | 794 | 86·5 | 83·9 | 88·8 |
| F | Vietnam | 598 | 3566 | 4164 | 14·4 | 13·3 | 15·5 |
| M | China | 130 | 3494 | 3624 | 3·6 | 3·0 | 4·2 |
| M | Hong Kong | 7934 | 5089 | 13023 | 60·9 | 60·1 | 61·8 |
| M | India | 14852 | 7088 | 21940 | 67·7 | 67·1 | 68·3 |
| M | Indonesia | 447 | 612 | 1059 | 42·2 | 39·2 | 45·2 |
| M | Korea | 821 | 373 | 1194 | 68·8 | 66·0 | 71·4 |
| M | Malaysia | 298 | 489 | 787 | 37·9 | 34·5 | 41·4 |
| M | Philippines | 2507 | 3318 | 5825 | 43·0 | 41·8 | 44·3 |
| M | Singapore | 121 | 164 | 285 | 42·5 | 36·6 | 48·4 |
| M | Taiwan | 1126 | 654 | 1780 | 63·3 | 61·0 | 65·5 |
| M | Thailand | 306 | 80 | 386 | 79·3 | 74·9 | 83·2 |
| M | Vietnam | 464 | 2484 | 2948 | 15·7 | 14·4 | 17·1 |

# **Supplementary Table 18.** Data table for education by dietitians at registration by countries/areas (Figure 3G in the main text)

| **Sex** | **Country/region** | **Yes** | **No** | **Total** | **Proportion** | **95% CI, lower** | **95% CI, upper** |
| --- | --- | --- | --- | --- | --- | --- | --- |
| F | China | 156 | 2703 | 2859 | 5·5 | 4·7 | 6·4 |
| F | Hong Kong | 7376 | 3193 | 10569 | 69·8 | 68·9 | 70·7 |
| F | India | 13761 | 2134 | 15895 | 86·6 | 86·0 | 87·1 |
| F | Indonesia | 328 | 607 | 935 | 35·1 | 32·0 | 38·2 |
| F | Korea | 678 | 319 | 997 | 68·0 | 65·0 | 70·9 |
| F | Malaysia | 416 | 304 | 720 | 57·8 | 54·1 | 61·4 |
| F | Philippines | 2761 | 5937 | 8698 | 31·7 | 30·8 | 32·7 |
| F | Singapore | 143 | 164 | 307 | 46·6 | 40·9 | 52·3 |
| F | Taiwan | 1056 | 418 | 1474 | 71·6 | 69·3 | 73·9 |
| F | Thailand | 556 | 237 | 793 | 70·1 | 66·8 | 73·3 |
| F | Vietnam | 744 | 3426 | 4170 | 17·8 | 16·7 | 19·0 |
| M | China | 191 | 3447 | 3638 | 5·3 | 4·5 | 6·0 |
| M | Hong Kong | 8362 | 4670 | 13032 | 64·2 | 63·3 | 65·0 |
| M | India | 19903 | 3055 | 22958 | 86·7 | 86·2 | 87·1 |
| M | Indonesia | 339 | 719 | 1058 | 32·0 | 29·2 | 34·9 |
| M | Korea | 808 | 388 | 1196 | 67·6 | 64·8 | 70·2 |
| M | Malaysia | 472 | 318 | 790 | 59·7 | 56·2 | 63·2 |
| M | Philippines | 2006 | 3839 | 5845 | 34·3 | 33·1 | 35·6 |
| M | Singapore | 133 | 148 | 281 | 47·3 | 41·4 | 53·3 |
| M | Taiwan | 1238 | 539 | 1777 | 69·7 | 67·5 | 71·8 |
| M | Thailand | 259 | 127 | 386 | 67·1 | 62·2 | 71·8 |
| M | Vietnam | 658 | 2296 | 2954 | 22·3 | 20·8 | 23·8 |

# **Supplementary Table 19.** Data table for education by podiatrists at registration by countries/areas (Figure 3H in the main text)

| **Sex** | **Country/region** | **Yes** | **No** | **Total** | **Proportion** | **95% CI, lower** | **95% CI, upper** |
| --- | --- | --- | --- | --- | --- | --- | --- |
| F | China | 826 | 2026 | 2852 | 29·0 | 27·3 | 30·7 |
| F | Hong Kong | 2014 | 8556 | 10570 | 19·1 | 18·3 | 19·8 |
| F | India | 7701 | 6129 | 13830 | 55·7 | 54·9 | 56·5 |
| F | Indonesia | 103 | 815 | 918 | 11·2 | 9·3 | 13·4 |
| F | Korea | 326 | 662 | 988 | 33·0 | 30·1 | 36·0 |
| F | Malaysia | 146 | 573 | 719 | 20·3 | 17·4 | 23·4 |
| F | Philippines | 86 | 8441 | 8527 | 1·0 | 0·8 | 1·2 |
| F | Singapore | 149 | 157 | 306 | 48·7 | 43·0 | 54·4 |
| F | Taiwan | 706 | 531 | 1237 | 57·1 | 54·3 | 59·9 |
| F | Thailand | 462 | 331 | 793 | 58·3 | 54·7 | 61·7 |
| F | Vietnam | 549 | 3571 | 4120 | 13·3 | 12·3 | 14·4 |
| M | China | 922 | 2706 | 3628 | 25·4 | 24·0 | 26·9 |
| M | Hong Kong | 2165 | 10841 | 13006 | 16·6 | 16·0 | 17·3 |
| M | India | 11075 | 8640 | 19715 | 56·2 | 55·5 | 56·9 |
| M | Indonesia | 117 | 924 | 1041 | 11·2 | 9·4 | 13·3 |
| M | Korea | 338 | 846 | 1184 | 28·5 | 26·0 | 31·2 |
| M | Malaysia | 209 | 578 | 787 | 26·6 | 23·5 | 29·8 |
| M | Philippines | 50 | 5689 | 5739 | 0·9 | 0·6 | 1·1 |
| M | Singapore | 144 | 135 | 279 | 51·6 | 45·6 | 57·6 |
| M | Taiwan | 840 | 704 | 1544 | 54·4 | 51·9 | 56·9 |
| M | Thailand | 217 | 166 | 383 | 56·7 | 51·5 | 61·7 |
| M | Vietnam | 446 | 2456 | 2902 | 15·4 | 14·1 | 16·7 |

# **Supplementary Table 20.** Data table for history of atherosclerotic cardiovascular disease (ASCVD) at registration by countries/areas (Figure 4A in the main text)

| **Sex** | **Country/region** | **Yes** | **No** | **Total** | **Proportion** | **95% CI, lower** | **95% CI, upper** |
| --- | --- | --- | --- | --- | --- | --- | --- |
| F | China | 660 | 2324 | 2984 | 22·1 | 20·6 | 23·7 |
| F | Hong Kong | 1726 | 8922 | 10648 | 16·2 | 15·5 | 16·9 |
| F | India | 2036 | 14484 | 16520 | 12·3 | 11·8 | 12·8 |
| F | Indonesia | 207 | 973 | 1180 | 17·5 | 15·4 | 19·8 |
| F | Korea | 112 | 895 | 1007 | 11·1 | 9·2 | 13·2 |
| F | Malaysia | 116 | 616 | 732 | 15·8 | 13·3 | 18·7 |
| F | Philippines | 724 | 8925 | 9649 | 7·5 | 7·0 | 8·0 |
| F | Singapore | 38 | 281 | 319 | 11·9 | 8·6 | 16·0 |
| F | Taiwan | 287 | 1191 | 1478 | 19·4 | 17·4 | 21·5 |
| F | Thailand | 76 | 720 | 796 | 9·5 | 7·6 | 11·8 |
| F | Vietnam | 407 | 4104 | 4511 | 9·0 | 8·2 | 9·9 |
| M | China | 791 | 3016 | 3807 | 20·8 | 19·5 | 22·1 |
| M | Hong Kong | 2907 | 10215 | 13122 | 22·2 | 21·4 | 22·9 |
| M | India | 3739 | 20228 | 23967 | 15·6 | 15·1 | 16·1 |
| M | Indonesia | 346 | 965 | 1311 | 26·4 | 24·0 | 28·9 |
| M | Korea | 169 | 1035 | 1204 | 14·0 | 12·1 | 16·1 |
| M | Malaysia | 243 | 553 | 796 | 30·5 | 27·3 | 33·9 |
| M | Philippines | 549 | 6065 | 6614 | 8·3 | 7·6 | 9·0 |
| M | Singapore | 65 | 230 | 295 | 22·0 | 17·4 | 27·2 |
| M | Taiwan | 465 | 1320 | 1785 | 26·1 | 24·0 | 28·2 |
| M | Thailand | 61 | 329 | 390 | 15·6 | 12·2 | 19·6 |
| M | Vietnam | 286 | 2975 | 3261 | 8·8 | 7·8 | 9·8 |

# **Supplementary Table 21.** Data table for history of heart failure hospitalization at registration by countries/areas (Figure 4B in the main text)

| **Sex** | **Country/region** | **Yes** | **No** | **Total** | **Proportion** | **95% CI, lower** | **95% CI, upper** |
| --- | --- | --- | --- | --- | --- | --- | --- |
| F | China | 6 | 2978 | 2984 | 0·2 | 0·1 | 0·4 |
| F | Hong Kong | 302 | 10346 | 10648 | 2·8 | 2·5 | 3·2 |
| F | India | 215 | 16305 | 16520 | 1·3 | 1·1 | 1·5 |
| F | Indonesia | 9 | 1171 | 1180 | 0·8 | 0·3 | 1·4 |
| F | Korea | 10 | 997 | 1007 | 1·0 | 0·5 | 1·8 |
| F | Malaysia | 12 | 720 | 732 | 1·6 | 0·8 | 2·8 |
| F | Philippines | 9 | 9640 | 9649 | 0·1 | 0·0 | 0·2 |
| F | Singapore | 1 | 318 | 319 | 0·3 | 0·0 | 1·7 |
| F | Taiwan | 29 | 1449 | 1478 | 2·0 | 1·3 | 2·8 |
| F | Thailand | 12 | 784 | 796 | 1·5 | 0·8 | 2·6 |
| F | Vietnam | 36 | 4475 | 4511 | 0·8 | 0·6 | 1·1 |
| M | China | 4 | 3803 | 3807 | 0·1 | 0·0 | 0·3 |
| M | Hong Kong | 367 | 12755 | 13122 | 2·8 | 2·5 | 3·1 |
| M | India | 716 | 23251 | 23967 | 3·0 | 2·8 | 3·2 |
| M | Indonesia | 53 | 1258 | 1311 | 4·0 | 3·0 | 5·3 |
| M | Korea | 18 | 1186 | 1204 | 1·5 | 0·9 | 2·4 |
| M | Malaysia | 18 | 778 | 796 | 2·3 | 1·3 | 3·6 |
| M | Philippines | 10 | 6604 | 6614 | 0·2 | 0·1 | 0·3 |
| M | Singapore | 5 | 290 | 295 | 1·7 | 0·6 | 3·9 |
| M | Taiwan | 51 | 1734 | 1785 | 2·9 | 2·1 | 3·7 |
| M | Thailand | 6 | 384 | 390 | 1·5 | 0·6 | 3·3 |
| M | Vietnam | 35 | 3226 | 3261 | 1·1 | 0·7 | 1·5 |

# **Supplementary Table 22.** Data table for estimated glomerular filtration (eGFR) <60 mL/min/1·73m^2^ at registration by countries/areas (Figure 4C in the main text)

| **Sex** | **Country/region** | **Yes** | **No** | **Total** | **Proportion** | **95% CI, lower** | **95% CI, upper** |
| --- | --- | --- | --- | --- | --- | --- | --- |
| F | China | 237 | 2571 | 2808 | 8·4 | 7·4 | 9·5 |
| F | Hong Kong | 1908 | 8704 | 10612 | 18·0 | 17·3 | 18·7 |
| F | India | 2921 | 9177 | 12098 | 24·1 | 23·4 | 24·9 |
| F | Indonesia | 175 | 457 | 632 | 27·7 | 24·2 | 31·4 |
| F | Korea | 176 | 752 | 928 | 19·0 | 16·5 | 21·6 |
| F | Malaysia | 131 | 547 | 678 | 19·3 | 16·4 | 22·5 |
| F | Philippines | 1300 | 2333 | 3633 | 35·8 | 34·2 | 37·4 |
| F | Singapore | 23 | 141 | 164 | 14·0 | 9·1 | 20·3 |
| F | Taiwan | 375 | 1068 | 1443 | 26·0 | 23·7 | 28·3 |
| F | Thailand | 279 | 497 | 776 | 36·0 | 32·6 | 39·4 |
| F | Vietnam | 918 | 3093 | 4011 | 22·9 | 21·6 | 24·2 |
| M | China | 195 | 3374 | 3569 | 5·5 | 4·7 | 6·3 |
| M | Hong Kong | 2422 | 10623 | 13045 | 18·6 | 17·9 | 19·2 |
| M | India | 2805 | 15150 | 17955 | 15·6 | 15·1 | 16·2 |
| M | Indonesia | 172 | 653 | 825 | 20·8 | 18·1 | 23·8 |
| M | Korea | 208 | 934 | 1142 | 18·2 | 16·0 | 20·6 |
| M | Malaysia | 225 | 536 | 761 | 29·6 | 26·3 | 32·9 |
| M | Philippines | 951 | 1998 | 2949 | 32·2 | 30·6 | 34·0 |
| M | Singapore | 32 | 134 | 166 | 19·3 | 13·6 | 26·1 |
| M | Taiwan | 448 | 1291 | 1739 | 25·8 | 23·7 | 27·9 |
| M | Thailand | 130 | 246 | 376 | 34·6 | 29·8 | 39·6 |
| M | Vietnam | 418 | 2490 | 2908 | 14·4 | 13·1 | 15·7 |

# **Supplementary Table 23.** Data table for any-site cancer at registration by countries/areas (Figure 4D in the main text)

| **Sex** | **Country/region** | **Yes** | **No** | **Total** | **Proportion** | **95% CI, lower** | **95% CI, upper** |
| --- | --- | --- | --- | --- | --- | --- | --- |
| F | China | 73 | 2911 | 2984 | 2·4 | 1·9 | 3·1 |
| F | Hong Kong | 669 | 9979 | 10648 | 6·3 | 5·8 | 6·8 |
| F | India | 86 | 16434 | 16520 | 0·5 | 0·4 | 0·6 |
| F | Indonesia | 15 | 1165 | 1180 | 1·3 | 0·7 | 2·1 |
| F | Korea | 55 | 952 | 1007 | 5·5 | 4·1 | 7·1 |
| F | Malaysia | 32 | 700 | 732 | 4·4 | 3·0 | 6·1 |
| F | Philippines | 108 | 9541 | 9649 | 1·1 | 0·9 | 1·3 |
| F | Singapore | 17 | 302 | 319 | 5·3 | 3·1 | 8·4 |
| F | Taiwan | 110 | 1368 | 1478 | 7·4 | 6·2 | 8·9 |
| F | Thailand | 9 | 787 | 796 | 1·1 | 0·5 | 2·1 |
| F | Vietnam | 51 | 4460 | 4511 | 1·1 | 0·8 | 1·5 |
| M | China | 38 | 3769 | 3807 | 1·0 | 0·7 | 1·4 |
| M | Hong Kong | 558 | 12564 | 13122 | 4·3 | 3·9 | 4·6 |
| M | India | 65 | 23902 | 23967 | 0·3 | 0·2 | 0·3 |
| M | Indonesia | 18 | 1293 | 1311 | 1·4 | 0·8 | 2·2 |
| M | Korea | 42 | 1162 | 1204 | 3·5 | 2·5 | 4·7 |
| M | Malaysia | 25 | 771 | 796 | 3·1 | 2·0 | 4·6 |
| M | Philippines | 27 | 6587 | 6614 | 0·4 | 0·3 | 0·6 |
| M | Singapore | 11 | 284 | 295 | 3·7 | 1·9 | 6·6 |
| M | Taiwan | 114 | 1671 | 1785 | 6·4 | 5·3 | 7·6 |
| M | Thailand | 4 | 386 | 390 | 1·0 | 0·3 | 2·6 |
| M | Vietnam | 19 | 3242 | 3261 | 0·6 | 0·4 | 0·9 |

# **Supplementary Table 24.** Cardiovascular risk profiles and quality of care among patients with type 2 diabetes in Asia, stratified by age group at registration

|  | **Age <50 years at registration** | | | | **Age ≥50 years at registration** | | | |
| --- | --- | --- | --- | --- | --- | --- | --- | --- |
|  | **n** | **Women (n=11,713)** | **n** | **Men**  **(n=17,128)** | **n** | **Women (n=38,037)** | **n** | **Men**  **(n=39,372)** |
| **Country/Area, n (%)** | 11,713 |  | 17,128 |  | 38,037 |  | 39,372 |  |
| China |  | 515 (4·4%) |  | 1,258 (7·3%) |  | 2,467 (6·5%) |  | 2,547 (6·5%) |
| Hong Kong |  | 1,928 (16·5%) |  | 2,823 (16·5%) |  | 8,720 (22·9%) |  | 10,299 (26·2%) |
| India |  | 5,852 (50·0%) |  | 9,053 (52·9%) |  | 10,655 (28·0%) |  | 14,892 (37·8%) |
| Indonesia |  | 229 (2·0%) |  | 391 (2·3%) |  | 951 (2·5%) |  | 920 (2·3%) |
| Korea |  | 156 (1·3%) |  | 263 (1·5%) |  | 851 (2·2%) |  | 941 (2·4%) |
| Malaysia |  | 146 (1·2%) |  | 140 (0·8%) |  | 586 (1·5%) |  | 656 (1·7%) |
| Philippines |  | 1,970 (16·8%) |  | 1,983 (11·6%) |  | 7,624 (20·0%) |  | 4,604 (11·7%) |
| Singapore |  | 77 (0·7%) |  | 71 (0·4%) |  | 242 (0·6%) |  | 224 (0·6%) |
| Taiwan |  | 98 (0·8%) |  | 241 (1·4%) |  | 1,380 (3·6%) |  | 1,544 (3·9%) |
| Thailand |  | 118 (1·0%) |  | 72 (0·4%) |  | 677 (1·8%) |  | 318 (0·8%) |
| Vietnam |  | 624 (5·3%) |  | 833 (4·9%) |  | 3,884 (10·2%) |  | 2,427 (6·2%) |
| College education, n (%) | 10,513 | 4,296 (40·9%) | 15,545 | 9,171 (59·0%) | 33,936 | 8,523 (25·1%) | 35,686 | 15,828 (44·4%) |
| Family history of diabetes, n (%) | 10,832 | 7,120 (65·7%) | 15,827 | 10,269 (64·9%) | 33,566 | 19,033 (56·7%) | 35,029 | 19,578 (55·9%) |
| Age, years | 11,713 | 42·3±6·8 | 17,128 | 42·2±6·4 | 38,037 | 63·0±8·4 | 39,372 | 61·9±8·1 |
| Duration of diabetes^¥^, years | 11,021 | 3·0 (1·0-7·0) | 16,198 | 3·0 (1·0-7·0) | 36,181 | 8·0 (3·0-14·0) | 37,638 | 8·0 (3·0-14·0) |
| Body mass index, kg/m^2^ | 10,742 | 27·1±5·3 | 15,938 | 26·8±4·5 | 34,545 | 26·0±4·7 | 36,752 | 25·8±4·0 |
| Waist circumference, cm | 8,996 | 88·4±13·3 | 13,745 | 92·3±11·7 | 28,671 | 88·3±11·9 | 32,022 | 92·0±11·0 |
| HbA_1c_, % | 9,321 | 8·2±2·0 | 14,152 | 8·3±2·1 | 31,447 | 7·9±1·8 | 34,114 | 7·9±1·8 |
| Systolic blood pressure, mmHg | 11,234 | 125·2±15·7 | 16,488 | 127·5±15·0 | 36,339 | 132·8±17·9 | 38,029 | 132·8±17·2 |
| Diastolic blood pressure, mmHg | 11,209 | 79·0±9·1 | 16,452 | 80·9±9·1 | 36,274 | 77·7±9·6 | 37,953 | 79·4±9·5 |
| Total cholesterol, mmol/L | 8,772 | 4·8±1·3 | 13,316 | 4·8±1·2 | 29,772 | 4·8±1·2 | 31,414 | 4·5±1·1 |
| Triglyceride^¥^, mmol/L | 9,087 | 1·6 (1·1-2·2) | 13,999 | 1·7 (1·2-2·4) | 30,753 | 1·5 (1·1-2·1) | 33,491 | 1·5 (1·0-2·1) |
| HDL-cholesterol, mmol/L | 8,948 | 1·1 (1·0, 1·4) | 13,625 | 1·0 (0·9, 1·2) | 30,206 | 1·2 (1·0, 1·5) | 32,822 | 1·1 (0·9, 1·3) |
| LDL-cholesterol, mmol/L | 8,870 | 2·7 (2·1, 3·3) | 13,504 | 2·7 (2·1, 3·3) | 30,050 | 2·6 (2·0, 3·3) | 32,646 | 2·5 (1·9, 3·1) |
| Non-HDL cholesterol, mmol/L | 8,460 | 3·4 (2·8, 4·2) | 12,757 | 3·5 (2·9, 4·3) | 28,694 | 3·3 (2·7, 4·1) | 30,250 | 3·2 (2·6, 3·9) |
| eGFR, mL/min/1·73m^2^ | 8,496 | 93·6±23·7 | 13,179 | 97·1±21·0 | 29,287 | 74·1±23·4 | 32,256 | 75·8±22·3 |
| **Cardiometabolic risk factors, n (%)** |  |  |  |  |  |  |  |  |
| Very high CVD risk^γ^ | 10,830 | 10,578 (97·7%) | 16,401 | 16,184 (98·7%) | 36,855 | 36,602 (99·3%) | 38,358 | 38,068 (99·2%) |
| HbA_1c_ <7% | 9,321 | 2,734 (29·3%) | 14,152 | 4,075 (28·8%) | 31,447 | 11,192 (35·6%) | 34,114 | 12,214 (35·8%) |
| Blood pressure <130/80 mmHg | 11,210 | 3,865 (34·5%) | 16,456 | 4,348 (26·4%) | 36,291 | 10,117 (27·9%) | 37,966 | 9,649 (25·4%) |
| Attained risk-based LDL-cholesterol target* | 8,777 | 1,356 (15·4%) | 13,408 | 2,130 (15·9%) | 29,926 | 4,932 (16·5%) | 32,514 | 6,495 (20·0%) |
| General obesity (BMI ≥25 kg/m^2^) | 10,742 | 6,566 (61·1%) | 15,938 | 10,187 (63·9%) | 34,545 | 18,235 (52·8%) | 36,752 | 20,249 (55·1%) |
| Central obesity (waist circumference ≥90 cm in men or ≥80 cm in women) | 8,996 | 6,751 (75·0%) | 13,745 | 7,895 (57·4%) | 28,671 | 22,426 (78·2%) | 32,022 | 18,350 (57·3%) |
| ≥3 treatment targets attained^#^ | 7,072 | 612 (8·7%) | 10,873 | 1,022 (9·4%) | 24,310 | 1,782 (7·3%) | 27,400 | 3,024 (11·0%) |
| Current smoker | 11,132 | 263 (2·4%) | 16,526 | 4,327 (26·2%) | 36,514 | 537 (1·5%) | 38,166 | 7,110 (18·6%) |
| Regular alcohol drinker | 11,117 | 66 (0·6%) | 16,493 | 1,757 (10·7%) | 36,472 | 99 (0·3%) | 38,060 | 2,739 (7·2%) |
| **Self-reported health habits in last 3 months, n (%)** | | | | | | | | |
| Adherence to balanced diet | 10,793 | 8,821 (81·7%) | 15,771 | 12,617 (80·0%) | 35,746 | 30,376 (85·0%) | 36,888 | 30,666 (83·1%) |
| Physical activity ≥3 times/week | 11,007 | 4,029 (36·6%) | 16,073 | 6,912 (43·0%) | 36,144 | 15,354 (42·5%) | 37,441 | 18,866 (50·4%) |
| SMBG ≥ once/week | 9,623 | 2,898 (30·1%) | 13,968 | 4,294 (30·7%) | 32,658 | 11,443 (35·0%) | 33,646 | 12,671 (37·7%) |
| **Exposure to allied health professionals, n(%)** | | | | | | | | |
| Education by nurses | 10,895 | 6,069 (55·7%) | 15,841 | 8,584 (54·2%) | 35,805 | 18,124 (50·6%) | 36,975 | 20,410 (55·2%) |
| Education by dietitians | 11,114 | 7,238 (65·1%) | 16,226 | 10,474 (64·6%) | 36,251 | 20,732 (57·2%) | 37,654 | 23,883 (63·4%) |
| Education by podiatrists | 10,150 | 3,240 (31·9%) | 14,757 | 4,929 (33·4%) | 34,659 | 9,824 (28·3%) | 35,416 | 11,587 (32·7%) |
| **Comorbidities, n (%)** | | | | | | | | |
| ASCVD | 11,713 | 635 (5·4%) | 17,128 | 1,196 (7·0%) | 38,037 | 5,752 (15·1%) | 39,372 | 8,423 (21·4%) |
| Heart failure | 11,713 | 19 (0·2%) | 17,128 | 74 (0·4%) | 38,037 | 622 (1·6%) | 39,372 | 1,209 (3·1%) |
| eGFR <60 mL/min/1·73m^2^ | 8,496 | 726 (8·5%) | 13,179 | 672 (5·1%) | 29,287 | 7,717 (26·3%) | 32,256 | 7,334 (22·7%) |
| Any-site cancer | 11,713 | 111 (0·9%) | 17,128 | 66 (0·4%) | 38,037 | 1,114 (2·9%) | 39,372 | 854 (2·2%) |
| **Medication use, n (%)** | | | | | | | | |
| Oral glucose-lowering drugs | 11,713 | 9,829 (83·9%) | 17,128 | 14,491 (84·6%) | 38,037 | 32,798 (86·2%) | 39,372 | 33,910 (86·1%) |
| Injectable GLP1-RA | 11,713 | 39 (0·3%) | 17,128 | 61 (0·4%) | 38,037 | 66 (0·2%) | 39,372 | 84 (0·2%) |
| Insulin | 11,713 | 2,773 (23·7%) | 17,128 | 3,593 (21·0%) | 38,037 | 10,684 (28·1%) | 39,372 | 11,386 (28·9%) |
| Blood pressure-lowering drugs | 11,713 | 4,179 (35·7%) | 17,128 | 6,512 (38·0%) | 38,037 | 25,055 (65·9%) | 39,372 | 25,259 (64·2%) |
| Renin-angiotensin system inhibitors | 10,382 | 2,228 (21·5%) | 15,176 | 3,541 (23·3%) | 31,673 | 13,719 (43·3%) | 33,436 | 14,928 (44·6%) |
| Statin | 10,239 | 2,602 (25·4%) | 14,712 | 4,532 (30·8%) | 31,746 | 14,356 (45·2%) | 33,165 | 14,983 (45·2%) |
| Aspirin | 11,713 | 460 (3·9%) | 17,128 | 1,052 (6·1%) | 38,037 | 5,886 (15·5%) | 39,372 | 8,262 (21·0%) |

Footnotes: Data are presented as mean ± standard deviation, ^¥^median (interquartile range) or number (percentage).

^γ^Definition of CV risk was based on the 2016 European Society of Cardiology/European Atherosclerosis Society (ESC/EAS) recommendations in line with the data collection period.^11,12^ ^#^We defined ≥3 treatment targets attained as 1) HbA_1c_<7%, 2) blood pressure <130/80 mmHg, 3) risk-based LDL-cholesterol target (<2·6 mmol/L if high risk or <1·8 mmol/L if very high-risk), and 4) lack of central obesity (waist circumference <90 cm in men or <80 cm in women).

ASCVD, atherosclerotic cardiovascular disease; BMI, body mass index; GLP1-RA, glucagon-like peptide 1 receptor analogues; HDL, high-density lipoprotein; LDL, low-density lipoprotein; SMBG, self-monitoring blood glucose.

# **Supplementary Table 25.** Data table for quality of diabetes care at registration among those with age ≥50 years at registration by countries/areas (Supplementary Figure 1A)

|  | **Sex** | **Yes** | **No** | **Total** | **Proportion** | **95% CI, lower** | **95% CI, upper** |
| --- | --- | --- | --- | --- | --- | --- | --- |
| Statin | F | 14356 | 17390 | 31746 | 45·2 | 44·7 | 45·8 |
| Statin | M | 14983 | 18182 | 33165 | 45·2 | 44·6 | 45·7 |
| RASi | F | 13719 | 17954 | 31673 | 43·3 | 42·8 | 43·9 |
| RASi | M | 14928 | 18508 | 33436 | 44·6 | 44·1 | 45·2 |
| HbA1c < 7% | F | 11192 | 20255 | 31447 | 35·6 | 35·1 | 36·1 |
| HbA1c < 7% | M | 12214 | 21900 | 34114 | 35·8 | 35·3 | 36·3 |
| BP < 130/80 mmHg | F | 10117 | 26174 | 36291 | 27·9 | 27·4 | 28·3 |
| BP < 130/80 mmHg | M | 9649 | 28317 | 37966 | 25·4 | 25·0 | 25·9 |
| LDL-cholesterol  < target | F | 4932 | 24994 | 29926 | 16·5 | 16·1 | 16·9 |
| LDL-cholesterol  < target | M | 6495 | 26019 | 32514 | 20·0 | 19·5 | 20·4 |
| BMI < 25 kg/m^2^ | F | 16310 | 18235 | 34545 | 47·2 | 46·7 | 47·7 |
| BMI < 25 kg/m^2^ | M | 16503 | 20249 | 36752 | 44·9 | 44·4 | 45·4 |
| Waist < 80cm | F | 6245 | 22426 | 28671 | 21·8 | 21·3 | 22·3 |
| Waist < 90cm | M | 13672 | 18350 | 32022 | 42·7 | 42·2 | 43·2 |
| Current smoker | F | 537 | 35977 | 36514 | 1·5 | 1·3 | 1·6 |
| Current smoker | M | 7110 | 31056 | 38166 | 18·6 | 18·2 | 19·0 |
| Regular drinker | F | 99 | 36373 | 36472 | 0·3 | 0·2 | 0·3 |
| Regular drinker | M | 2739 | 35321 | 38060 | 7·2 | 6·9 | 7·5 |
| SMBG | F | 11443 | 21215 | 32658 | 35·0 | 34·5 | 35·6 |
| SMBG | M | 12671 | 20975 | 33646 | 37·7 | 37·1 | 38·2 |
| Regular exercise | F | 15354 | 20790 | 36144 | 42·5 | 42·0 | 43·0 |
| Regular exercise | M | 18866 | 18575 | 37441 | 50·4 | 49·9 | 50·9 |

Footnotes: Risk-based LDL-cholesterol target (<2·6 mmol/L if high risk or <1·8 mmol/L if very high-risk) was defined by 2016 European Society of Cardiology/European Atherosclerosis Society (ESC/EAS) recommendations in line with the data collection period.^11,12^ RASi: renin angiotensin system inhibitor; SMBG: self-monitoring of blood glucose.

# **Supplementary Table 26.** Data table for quality of diabetes care at registration among those with a history of ASCVD and/or heart failure at registration by countries/areas (Supplementary Figure 1B)

|  | **Sex** | **Yes** | **No** | **Total** | **Proportion** | **95% CI, lower** | **95% CI, upper** |
| --- | --- | --- | --- | --- | --- | --- | --- |
| Statin | F | 3216 | 2074 | 5290 | 60·8 | 59·5 | 62·1 |
| Statin | M | 5422 | 2525 | 7947 | 68·2 | 67·2 | 69·3 |
| RASi | F | 3068 | 2192 | 5260 | 58·3 | 57·0 | 59·7 |
| RASi | M | 4957 | 3229 | 8186 | 60·6 | 59·5 | 61·6 |
| HbA1c < 7% | F | 1738 | 3933 | 5671 | 30·6 | 29·4 | 31·9 |
| HbA1c < 7% | M | 2990 | 5965 | 8955 | 33·4 | 32·4 | 34·4 |
| BP < 130/80mmHg | F | 1549 | 4902 | 6451 | 24·0 | 23·0 | 25·1 |
| BP < 130/80mmHg | M | 2295 | 7405 | 9700 | 23·7 | 22·8 | 24·5 |
| LDL-cholesterol  < target | F | 1167 | 4407 | 5574 | 20·9 | 19·9 | 22·0 |
| LDL-cholesterol  < target | M | 2107 | 6635 | 8742 | 24·1 | 23·2 | 25·0 |
| BMI < 25kg/m^2^ | F | 2791 | 3527 | 6318 | 44·2 | 42·9 | 45·4 |
| BMI < 25 kg/m^2^ | M | 3848 | 5736 | 9584 | 40·2 | 39·2 | 41·1 |
| Waist < 80cm | F | 1184 | 4418 | 5602 | 21·1 | 20·1 | 22·2 |
| Waist < 90cm | M | 3636 | 5189 | 8825 | 41·2 | 40·2 | 42·2 |
| Current smoker | F | 97 | 6374 | 6471 | 1·5 | 1·2 | 1·8 |
| Current smoker | M | 1691 | 8055 | 9746 | 17·4 | 16·6 | 18·1 |
| Regular drinker | F | 14 | 6463 | 6477 | 0·2 | 0·1 | 0·4 |
| Regular drinker | M | 657 | 9090 | 9747 | 6·7 | 6·3 | 7·3 |
| SMBG | F | 2421 | 3463 | 5884 | 41·1 | 39·9 | 42·4 |
| SMBG | M | 3955 | 4907 | 8862 | 44·6 | 43·6 | 45·7 |
| Regular exercise | F | 2500 | 3927 | 6427 | 38·9 | 37·7 | 40·1 |
| Regular exercise | M | 4690 | 4940 | 9630 | 48·7 | 47·7 | 49·7 |

Footnotes: Risk-based LDL-cholesterol target (<2·6 mmol/L if high risk or <1·8 mmol/L if very high-risk) was defined by 2016 European Society of Cardiology/European Atherosclerosis Society (ESC/EAS) recommendations in line with the data collection period.^11,12^ RASi: renin angiotensin system inhibitor; SMBG: self-monitoring of blood glucose.

# **Supplementary Table 27.** Data table for quality of diabetes care at registration among those with a history of eGFR <60 mL/min/1·73m^2^ at registration by countries/areas (Supplementary Figure 1C)

|  | **Sex** | **Yes** | **No** | **Total** | **Proportion** | **95% CI, lower** | **95% CI, upper** |
| --- | --- | --- | --- | --- | --- | --- | --- |
| Statin | F | 3906 | 3217 | 7123 | 54·8 | 53·7 | 56·0 |
| Statin | M | 3878 | 3011 | 6889 | 56·3 | 55·1 | 57·5 |
| RASi | F | 3996 | 3021 | 7017 | 56·9 | 55·8 | 58·1 |
| RASi | M | 4248 | 2673 | 6921 | 61·4 | 60·2 | 62·5 |
| HbA1c < 7% | F | 2480 | 5169 | 7649 | 32·4 | 31·4 | 33·5 |
| HbA1c < 7% | M | 2538 | 4854 | 7392 | 34·3 | 33·3 | 35·4 |
| BP < 130/80 mmHg | F | 1831 | 6449 | 8280 | 22·1 | 21·2 | 23·0 |
| BP < 130/80 mmHg | M | 1714 | 6179 | 7893 | 21·7 | 20·8 | 22·6 |
| LDL-cholesterol  < target | F | 1402 | 5989 | 7391 | 19·0 | 18·1 | 19·9 |
| LDL-cholesterol  < target | M | 1556 | 5417 | 6973 | 22·3 | 21·3 | 23·3 |
| BMI < 25 kg/m^2^ | F | 3470 | 4450 | 7920 | 43·8 | 42·7 | 44·9 |
| BMI < 25 kg/m^2^ | M | 3253 | 4372 | 7625 | 42·7 | 41·5 | 43·8 |
| Waist < 80cm | F | 1391 | 5460 | 6851 | 20·3 | 19·4 | 21·3 |
| Waist < 90cm | M | 2825 | 3977 | 6802 | 41·5 | 40·4 | 42·7 |
| Current smoker | F | 112 | 8071 | 8183 | 1·4 | 1·1 | 1·6 |
| Current smoker | M | 1116 | 6779 | 7895 | 14·1 | 13·4 | 14·9 |
| Regular drinker | F | 19 | 8169 | 8188 | 0·2 | 0·1 | 0·4 |
| Regular drinker | M | 333 | 7538 | 7871 | 4·2 | 3·8 | 4·7 |
| SMBG | F | 2866 | 4700 | 7566 | 37·9 | 36·8 | 39·0 |
| SMBG | M | 3087 | 4002 | 7089 | 43·5 | 42·4 | 44·7 |
| Regular exercise | F | 3101 | 5094 | 8195 | 37·8 | 36·8 | 38·9 |
| Regular exercise | M | 3843 | 3973 | 7816 | 49·2 | 48·1 | 50·3 |

Footnotes: Risk-based LDL-cholesterol target (<2·6 mmol/L if high risk or <1·8 mmol/L if very high-risk) was defined by 2016 European Society of Cardiology/European Atherosclerosis Society (ESC/EAS) recommendations in line with the data collection period.^11,12^ RASi: renin angiotensin system inhibitor; SMBG: self-monitoring of blood glucose.

# **Supplementary Table 28.** Cardiovascular risk profiles and quality of care among male patients with type 2 diabetes who were either included or excluded (due to missing data) in the analysis

|  | **Included (N=25,376)** | | **Excluded (N=31,176)** | |
| --- | --- | --- | --- | --- |
|  | **n** |  | **n** |  |
| **Country/Area, n (%)** | 25,376 |  | 31,176 |  |
| China |  | 1,925 (7·6%) |  | 1,882 (6·0%) |
| Hong Kong |  | 10,419 (41·1%) |  | 2,703 (8·7%) |
| India |  | 8,280 (32·6%) |  | 15,687 (50·3%) |
| Indonesia |  | 376 (1·5%) |  | 935 (3·0%) |
| Korea |  | 606 (2·4%) |  | 598 (1·9%) |
| Malaysia |  | 544 (2·1%) |  | 252 (0·8%) |
| Philippines |  | 218 (0·9%) |  | 6,396 (20·5%) |
| Singapore |  | 113 (0·4%) |  | 182 (0·6%) |
| Taiwan |  | 1,233 (4·9%) |  | 552 (1·8%) |
| Thailand |  | 284 (1·1%) |  | 106 (0·3%) |
| Vietnam |  | 1,378 (5·4%) |  | 1,883 (6·0%) |
| College education, n (%) | 25,376 | 10,148 (40·0%) | 25,884 | 14,868 (57·4%) |
| Family history of diabetes, n (%) | 23,620 | 13,762 (58·3%) | 27,277 | 16,105 (59·0%) |
| Age, years | 25,376 | 57·0±11·9 | 31,124 | 55·1±11·7 |
| Duration of diabetes^¥^, years | 25,376 | 6·00 (2·00, 12·0) | 28,480 | 5·00 (2·00, 11·0) |
| Body mass index, kg/m^2^ | 25,280 | 26·1±4·06 | 27,427 | 26·1±4·36 |
| Waist circumference, cm | 25,376 | 92·2±10·9 | 20,403 | 91·9±11·6 |
| HbA_1c_, % | 25,376 | 7·91±1·81 | 22,909 | 8·12±2·0 |
| Systolic blood pressure, mmHg | 25,376 | 132·0±16·8 | 29,177 | 130·0±16·7 |
| Diastolic blood pressure, mmHg | 25,372 | 79·5±9·60 | 29,069 | 80·2±9·17 |
| Total cholesterol, mmol/L | 23,618 | 4·40 (3·80, 5·10) | 21,127 | 4·53 (3·89, 5·30) |
| Triglyceride^¥^, mmol/L | 25,190 | 1·49 (1·01, 2·08) | 22,317 | 1·67 (1·19, 2·30) |
| HDL-cholesterol, mmol/L | 25,069 | 1·10 (0·95, 1·30) | 21,394 | 1·06 (0·93, 1·24) |
| LDL-cholesterol, mmol/L | 25,376 | 2·50 (1·94, 3·07) | 20,786 | 2·60 (2·02, 3·20) |
| Non-HDL cholesterol, mmol/L | 23,447 | 3·21 (2·65, 3·94) | 19,572 | 3·39 (2·77, 4·17) |
| eGFR, mL/min/1·73m^2^ | 25,376 | 82·0±23·7 | 20059 | 82·0±24·3 |
| **Cardiometabolic risk factors, n (%)** | | | | |
| Very high CVD risk^γ^ | 25,376 | 24,953 (98·3%) | 29,419 | 29,335 (99·7%) |
| HbA_1c_ <7% | 25,376 | 8,918 (35·1%) | 22,909 | 7,380 (32·2%) |
| Blood pressure <130/80 mmHg | 25,376 | 7,259 (28·6%) | 29,082 | 6,746 (23·2%) |
| Attained risk-based LDL-cholesterol target* | 25,376 | 5,075 (20·0%) | 20,558 | 3,552 (17·3%) |
| General obesity (BMI ≥25 kg/m^2^) | 25,280 | 14,467 (57·2%) | 27,427 | 15,980 (58·3%) |
| Central obesity (waist circumference ≥90 cm in men or ≥80 cm in women) | 25,376 | 14,745 (58·1%) | 20,403 | 11,506 (56·4%) |
| ≥3 treatment targets attained^#^ | 25,376 | 2,819 (11·1%) | 12,901 | 1,227 (9·5%) |
| Current smoker | 25,376 | 5,194 (20·5%) | 29,358 | 6,249 (21·3%) |
| Regular alcohol drinker | 25,376 | 1,831 (7·2%) | 29,220 | 2,670 (9·1%) |
| **Self-reported health habits in the last 3 months, n (%)** | | | | |
| Adherence to balanced diet | 25,376 | 21,634 (85·3%) | 27,316 | 21,662 (79·3%) |
| Physical activity ≥3 times/week | 25,376 | 12,245 (48·3%) | 28,172 | 13,558 (48·1%) |
| SMBG ≥ once/week | 25,376 | 10,158 (40·0%) | 22,268 | 6,825 (30·6%) |
| **Ever exposure to allied health professionals, n (%)** | | | | |
| Education by nurses | 24,797 | 13,945 (56·2%) | 28,054 | 15,061 (53·7%) |
| Education by dietitians | 25,229 | 16,666 (66·1%) | 28,686 | 17,703 (61·7%) |
| Education by podiatrists | 24,011 | 8,047 (33·5%) | 26,197 | 8,476 (32·4%) |
| **Comorbidities, n (%)** |  |  |  |  |
| ASCVD | 25,376 | 5,039 (19·9%) | 31,176 | 4,582 (14·7%) |
| Heart failure | 25,376 | 702 (2·8%) | 31,176 | 581 (1·9%) |
| eGFR <60 mL/min/1·73m^2^ | 25,376 | 4,455 (17·6%) | 20,059 | 3,551 (17·7%) |
| Any-site cancer | 25,376 | 688 (2·7%) | 31,176 | 233 (0·7%) |
| **Medication use, n (%)** |  |  |  |  |
| Oral glucose-lowering drugs | 25,376 | 22,239 (87·6%) | 31,176 | 26,182 (84·0%) |
| Injectable GLP1-RA | 25,376 | 83 (0·3%) | 31,176 | 62 (0·2%) |
| Insulin | 25,376 | 7,200 (28·4%) | 31,176 | 7,787 (25·0%) |
| Blood pressure-lowering drugs | 25,376 | 15,274 (60·2%) | 31,176 | 16,504 (52·9%) |
| Renin-angiotensin system inhibitors | 23,805 | 10,696 (44·9%) | 24,856 | 7,776 (31·3%) |
| Statin | 25,376 | 12,026 (47·4%) | 22,550 | 7,492 (33·2%) |
| Aspirin | 25,376 | 5,756 (22·7%) | 31,176 | 3,559 (11·4%) |

Footnotes: Data are presented as mean ± standard deviation, ^¥^median (interquartile range) or number (percentage)·

^γ^Definition of CV risk was based on the 2016 European Society of Cardiology/European Atherosclerosis Society (ESC/EAS) recommendations in line with the data collection period·^11,12^ ^#^We defined ≥3 treatment targets attained as 1) HbA_1c_<7%, 2) blood pressure <130/80 mmHg, 3) risk-based LDL-cholesterol target (<2·6 mmol/L if high risk or <1·8 mmol/L if very high-risk), and 4) lack of central obesity (waist circumference <90 cm in men or <80 cm in women)·

ASCVD, atherosclerotic cardiovascular disease; BMI, body mass index; GLP1-RA, glucagon-like peptide 1 receptor analogues; HDL, high-density lipoprotein; LDL, low-density lipoprotein; SMBG, self-monitoring blood glucose·

# **Supplementary Table 29.** Cardiovascular risk profiles and quality of care among female patients with type 2 diabetes who were either included or excluded (due to missing data) in the analysis

|  | **Included (N=20,784)** | | **Excluded (N=29,040)** | |
| --- | --- | --- | --- | --- |
|  | **n** |  | **n** |  |
| **Country/Area, n (%)** | 20,784 |  | 29,040 |  |
| China |  | 1,504 (7·2%) |  | 1,480 (5·1%) |
| Hong Kong |  | 8,575 (41·3%) |  | 2,073 (7·1%) |
| India |  | 5,499 (26·5%) |  | 11,021 (38·0%) |
| Indonesia |  | 297 (1·4%) |  | 883 (3·0%) |
| Korea |  | 479 (2·3%) |  | 528 (1·8%) |
| Malaysia |  | 457 (2·2%) |  | 275 (0·9%) |
| Philippines |  | 272 (1·3%) |  | 9,377 (32·3%) |
| Singapore |  | 117 (0·6%) |  | 202 (0·7%) |
| Taiwan |  | 1,036 (5·0%) |  | 442 (1·5%) |
| Thailand |  | 626 (3·0%) |  | 170 (0·6%) |
| Vietnam |  | 1,922 (9·2%) |  | 2,589 (8·9%) |
| College education, n (%) | 20,784 | 21·0% (4357) | 23,701 | 8,481 (35·8%) |
| Family history of diabetes, n (%) | 19,336 | 11,470 (59·3%) | 25,110 | 14,710 (58·6%) |
| Age, years | 20,784 | 58·7±12·0 | 28,966 | 57·7±11·9 |
| Duration of diabetes^¥^, years | 20,784 | 7·00 (3·00, 13·0) | 26,452 | 6·00 (2·00, 11·0) |
| Body mass index, kg/m^2^ | 20,690 | 26·3±4·81 | 24,608 | 26·2±4·96 |
| Waist circumference, cm | 20,784 | 88·5±11·8 | 16,892 | 88·1±12·8 |
| HbA_1c_, % | 20,784 | 7·87±1·72 | 20,022 | 8·08±2·01 |
| Systolic blood pressure, mmHg | 20,784 | 133·0±17·9 | 26,847 | 129·0±17·4 |
| Diastolic blood pressure, mmHg | 20,782 | 77·3±9·67 | 26,759 | 78·5±9·37 |
| Total cholesterol, mmol/L | 19,827 | 4·60 (4·00, 5·30) | 18,749 | 4·74 (4·04, 5·57) |
| Triglyceride^¥^, mmol/L | 20,649 | 1·50 (1·08, 2·03) | 19,221 | 1·62 (1·19, 2·20) |
| HDL-cholesterol, mmol/L | 20,570 | 1·20 (1·04, 1·50) | 18,614 | 1·17 (1·00, 1·40) |
| LDL-cholesterol, mmol/L | 20,784 | 2·54 (2·00, 3·13) | 18,166 | 2·70 (2·11, 3·40) |
| Non-HDL cholesterol, mmol/L | 19,704 | 3·29 (2·70, 4·00) | 17,478 | 3·48 (2·80, 4·29) |
| eGFR, mL/min/1·73m^2^ | 20,784 | 80·2±24·4 | 16,999 | 76·4±25·1 |
| **Cardiometabolic risk factors, n (%)** | | | | |
| Very high CVD risk^γ^ | 20,784 | 20,375 (98·0%) | 26,953 | 26,857 (99·6%) |
| HbA_1c_ <7% | 20,784 | 7,189 (34·6%) | 20,022 | 6,751 (33·7%) |
| Blood pressure <130/80 mmHg | 20,784 | 6,450 (31·0%) | 26,775 | 7,554 (28·2%) |
| Attained risk-based LDL-cholesterol target* | 20,784 | 3,646 (17·5%) | 17,946 | 2,646 (14·7%) |
| General obesity (BMI ≥25 kg/m^2^) | 20,690 | 11,476 (55·5%) | 24,608 | 13,331 (54·2%) |
| Central obesity (waist circumference ≥90 cm in men or ≥80 cm in women) | 20,784 | 16,368 (78·8%) | 16,892 | 12,816 (75·9%) |
| ≥3 treatment targets attained^#^ | 20,784 | 1,650 (7·9%) | 10,604 | 744 (7·0%) |
| Current smoker | 20,784 | 333 (1·6%) | 26,919 | 467 (1·7%) |
| Regular alcohol drinker | 20,784 | 67 (0·3%) | 26,861 | 98 (0·4%) |
| **Self-reported health habits in the last 3 months, n (%)** | | | | |
| Adherence to balanced diet | 20,784 | 18,265 (87·9%) | 25,806 | 20,942 (81·2%) |
| Physical activity ≥3 times/week | 20,784 | 9,388 (45·2%) | 26,417 | 10,039 (38·0%) |
| SMBG ≥ once/week | 20,784 | 8,008 (38·5%) | 21,546 | 6,372 (29·6%) |
| **Ever exposure to allied health professionals, n (%)** | | | | |
| Education by nurses | 20,433 | 11,489 (56·2%) | 26,318 | 12,710 (48·3%) |
| Education by dietitians | 20,648 | 13,533 (65·5%) | 26,769 | 14,442 (54·0%) |
| Education by podiatrists | 19,835 | 6,469 (32·6%) | 25,025 | 6,599 (26·4%) |
| **Comorbidities, n (%)** |  |  |  |  |
| ASCVD | 20,784 | 3,069 (14·8%) | 29,040 | 3,320 (11·4%) |
| Heart failure | 20,784 | 376 (1·8%) | 29,040 | 265 (0·9%) |
| eGFR <60 mL/min/1·73m^2^ | 20,784 | 4,231 (20·4%) | 16,999 | 4,212 (24·8%) |
| Any-site cancer | 20,784 | 782 (3·8%) | 29,040 | 443 (1·5%) |
| **Medication use, n (%)** |  |  |  |  |
| Oral glucose-lowering drugs | 20,784 | 18,414 (88·6%) | 29,040 | 24,230 (83·4%) |
| Injectable GLP1-RA | 20,784 | 61 (0·3%) | 29,040 | 44 (0·2%) |
| Insulin | 20,784 | 6·149 (29·6%) | 29,040 | 7,310 (25·2%) |
| Blood pressure-lowering drugs | 20,784 | 13,051 (62·8%) | 29,040 | 16,200 (55·8%) |
| Renin-angiotensin system inhibitors | 19,370 | 8,644 (44·6%) | 22,753 | 7,310 (32·1%) |
| Statin | 20,784 | 10,100 (48·6%) | 21,274 | 6,863 (32·3%) |
| Aspirin | 20,784 | 3,871 (18·6%) | 29,040 | 2,479 (8·5%) |

Footnotes: Data are presented as mean ± standard deviation, ^¥^median (interquartile range) or number (percentage)·

^γ^Definition of CV risk was based on the 2016 European Society of Cardiology/European Atherosclerosis Society (ESC/EAS) recommendations in line with the data collection period·^11,12^ ^#^We defined ≥3 treatment targets attained as 1) HbA_1c_<7%, 2) blood pressure <130/80 mmHg, 3) risk-based LDL-cholesterol target (<2·6 mmol/L if high risk or <1·8 mmol/L if very high-risk), and 4) lack of central obesity (waist circumference <90 cm in men or <80 cm in women)·

ASCVD, atherosclerotic cardiovascular disease; BMI, body mass index; GLP1-RA, glucagon-like peptide 1 receptor analogues; HDL, high-density lipoprotein; LDL, low-density lipoprotein; SMBG, self-monitoring blood glucose·

# **Supplementary Figure 1.** Quality of diabetes care at registration, stratified by age and history of comorbidities estimated by proportions with 95% estimates

1. **Age ≥50 years**


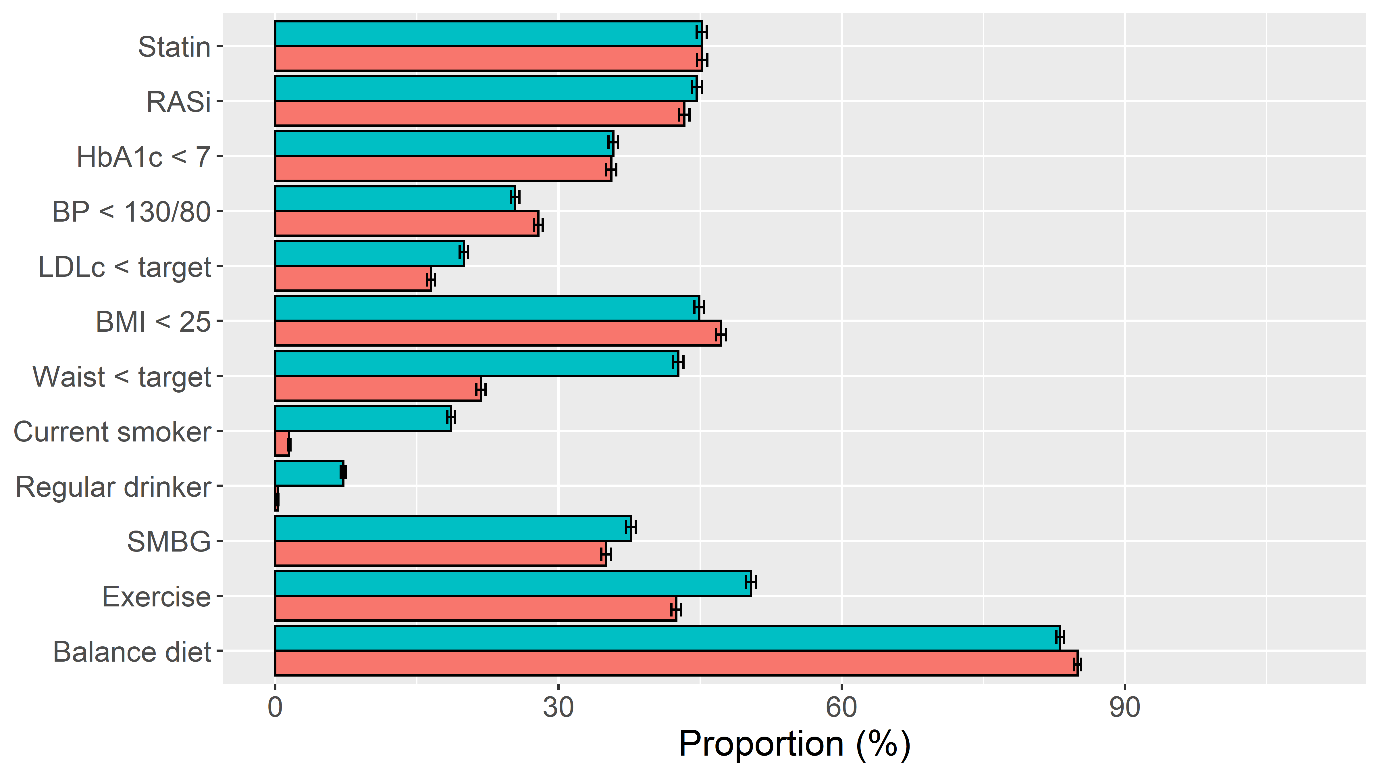


1. **History of ASCVD and/or heart failure**


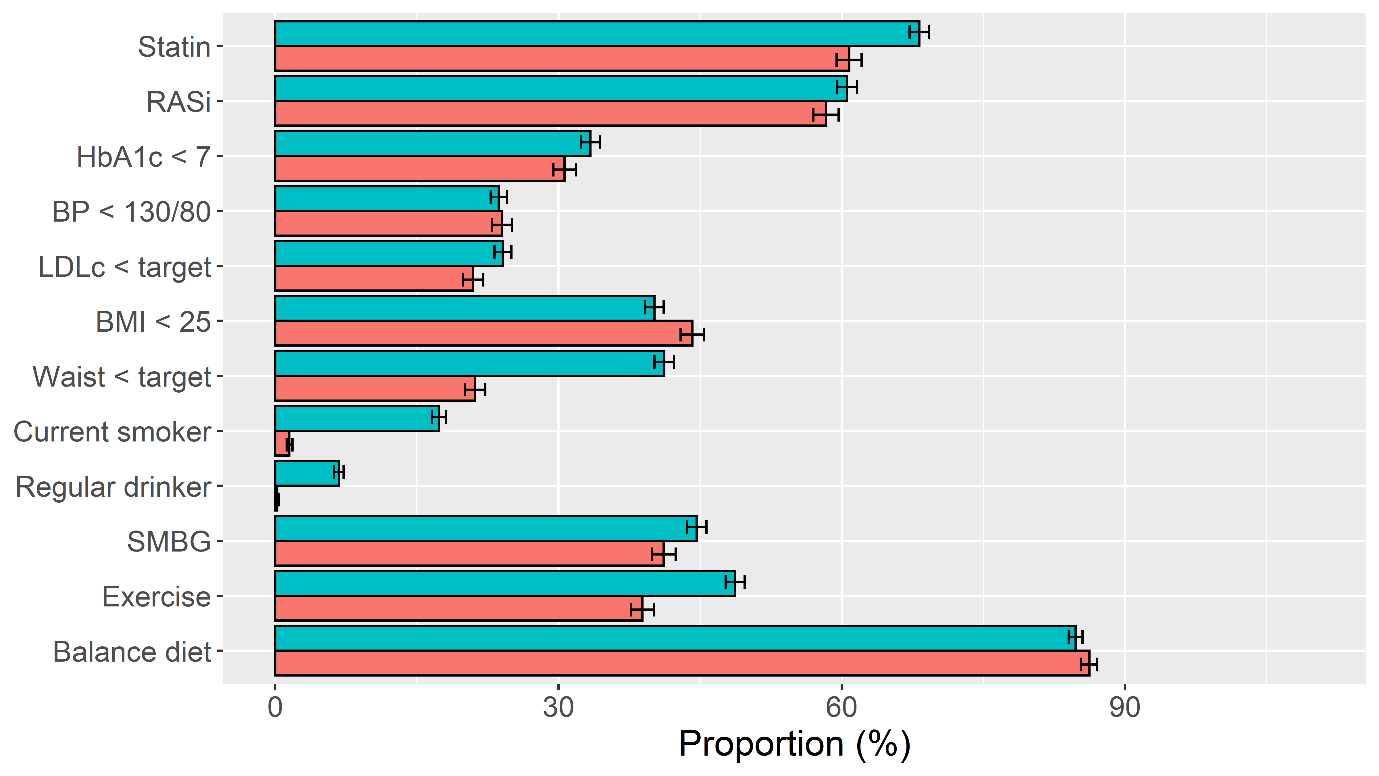


1. **History of eGFR <60 mL/min/1·73m^2^**


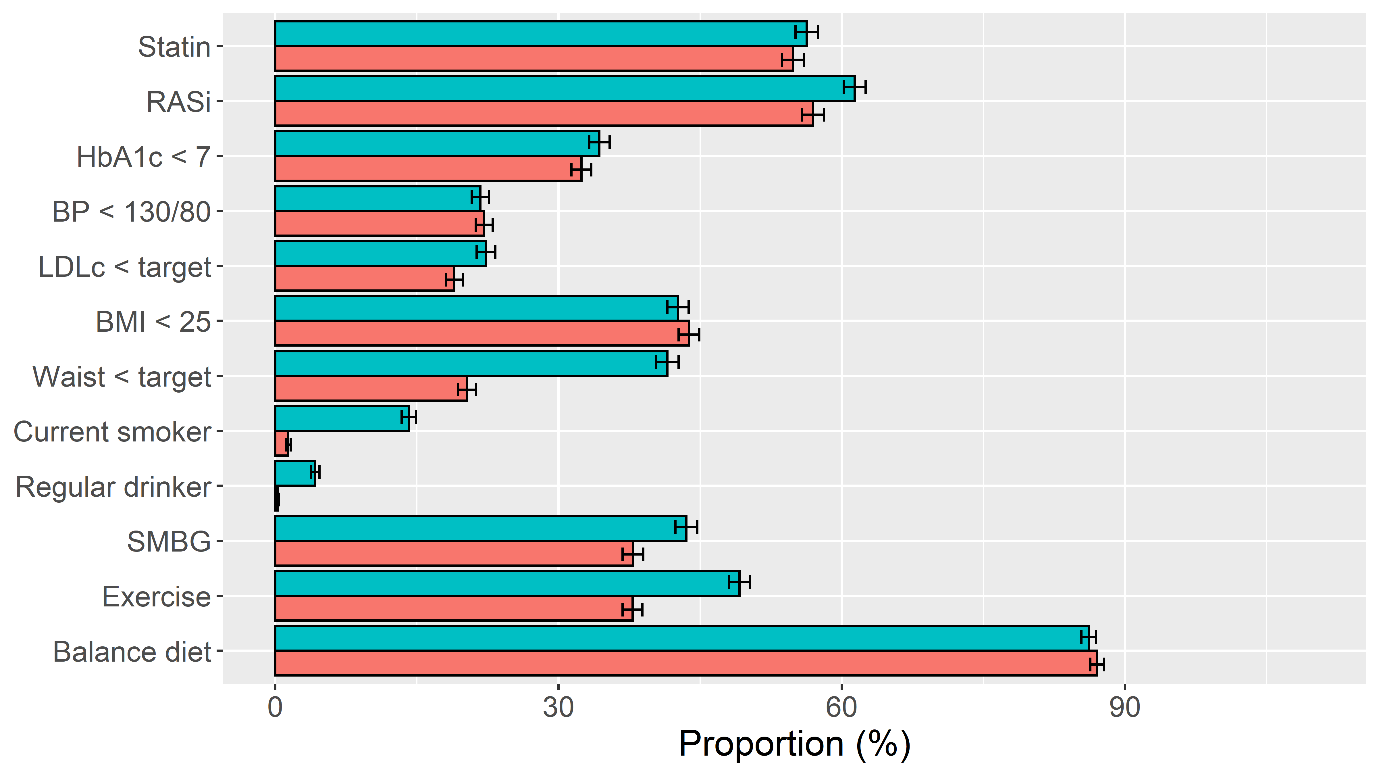


Footnotes: Green bars represent men· Orange bars represent women.

^γ^Definition of CV risk was based on the 2016 European Society of Cardiology/European Atherosclerosis Society (ESC/EAS) recommendations in line with the data collection period. ^#^Risk-based LDL-cholesterol target was defined as <2·6 mmol/L if high risk or <1·8 mmol/L if very high-risk. General obesity was defined as body mass index ≥25 kg/m^2^. Waist circumference target was defined as <90 cm in men or <80 cm in women.

ASCVD, atherosclerotic cardiovascular disease; BP, blood pressure; eGFR, estimated glomerular filtration rate (creatinine-based CKD-EPI formula); LDLc, low-density lipoprotein cholesterol (in mmol/L).

# **References**

1. Chan JCN, Lim LL, Luk AOY, et al. From Hong Kong Diabetes Register to JADE Program to RAMP-DM for Data-Driven Actions. *Diabetes Care* 2019; **42**(11): 2022-31.

2. Lim LL, Lau ESH, Kong APS, et al. Aspects of Multicomponent Integrated Care Promote Sustained Improvement in Surrogate Clinical Outcomes: A Systematic Review and Meta-analysis. *Diabetes Care* 2018; **41**(6): 1312-20.

3. Chan JCN, Lim LL, Wareham NJ, et al. The Lancet Commission on diabetes: using data to transform diabetes care and patient lives. *Lancet* 2021; **396**(10267): 2019-82.

4. Lim LL, Lau ESH, Ozaki R, et al. Association of technologically assisted integrated care with clinical outcomes in type 2 diabetes in Hong Kong using the prospective JADE Program: A retrospective cohort analysis. *PLoS Med* 2020; **17**(10): e1003367.

5. Wu H, Lau ESH, Ma RCW, et al. Secular trends in all-cause and cause-specific mortality rates in people with diabetes in Hong Kong, 2001-2016: a retrospective cohort study. *Diabetologia* 2020; **63**(4): 757-66.

6. Ko GT, So WY, Tong PC, et al. From design to implementation--the Joint Asia Diabetes Evaluation (JADE) program: a descriptive report of an electronic web-based diabetes management program. *BMC Med Inform Decis Mak* 2010; **10**: 26.

7. Lim LL, Lau ESH, Fu AWC, et al. Effects of a Technology-Assisted Integrated Diabetes Care Program on Cardiometabolic Risk Factors Among Patients With Type 2 Diabetes in the Asia-Pacific Region: The JADE Program Randomized Clinical Trial. *JAMA Netw Open* 2021; **4**(4): e217557.

8. Chan JCN, Thewjitcharoen Y, Nguyen TK, et al. Effect of a Web-Based Management Guide on Risk Factors in Patients With Type 2 Diabetes and Diabetic Kidney Disease: A JADE Randomized Clinical Trial. *JAMA Netw Open* 2022; **5**(3): e223862.

9. Association AD. 5. Glycemic Targets. *Diabetes Care* 2015; **39**(Supplement_1): S39-S46.

10. Association AD. 8. Cardiovascular Disease and Risk Management. *Diabetes Care* 2015; **39**(Supplement_1): S60-S71.

11. Catapano AL, Graham I, De Backer G, et al. 2016 ESC/EAS Guidelines for the Management of Dyslipidaemias. *Eur Heart J* 2016; **37**(39): 2999-3058.

12. Reiner Z, Catapano AL, De Backer G, et al. ESC/EAS Guidelines for the management of dyslipidaemias: the Task Force for the management of dyslipidaemias of the European Society of Cardiology (ESC) and the European Atherosclerosis Society (EAS). *Eur Heart J* 2011; **32**(14): 1769-818.
